# Supplementary figures and images for: Autophagy compensates for defects in mitochondrial dynamics
Source: PLoS Genet. 2020 Mar 19;16(3):e1008638. doi: 10.1371/journal.pgen.1008638 (PMC7135339; doi:10.1371/journal.pgen.1008638)

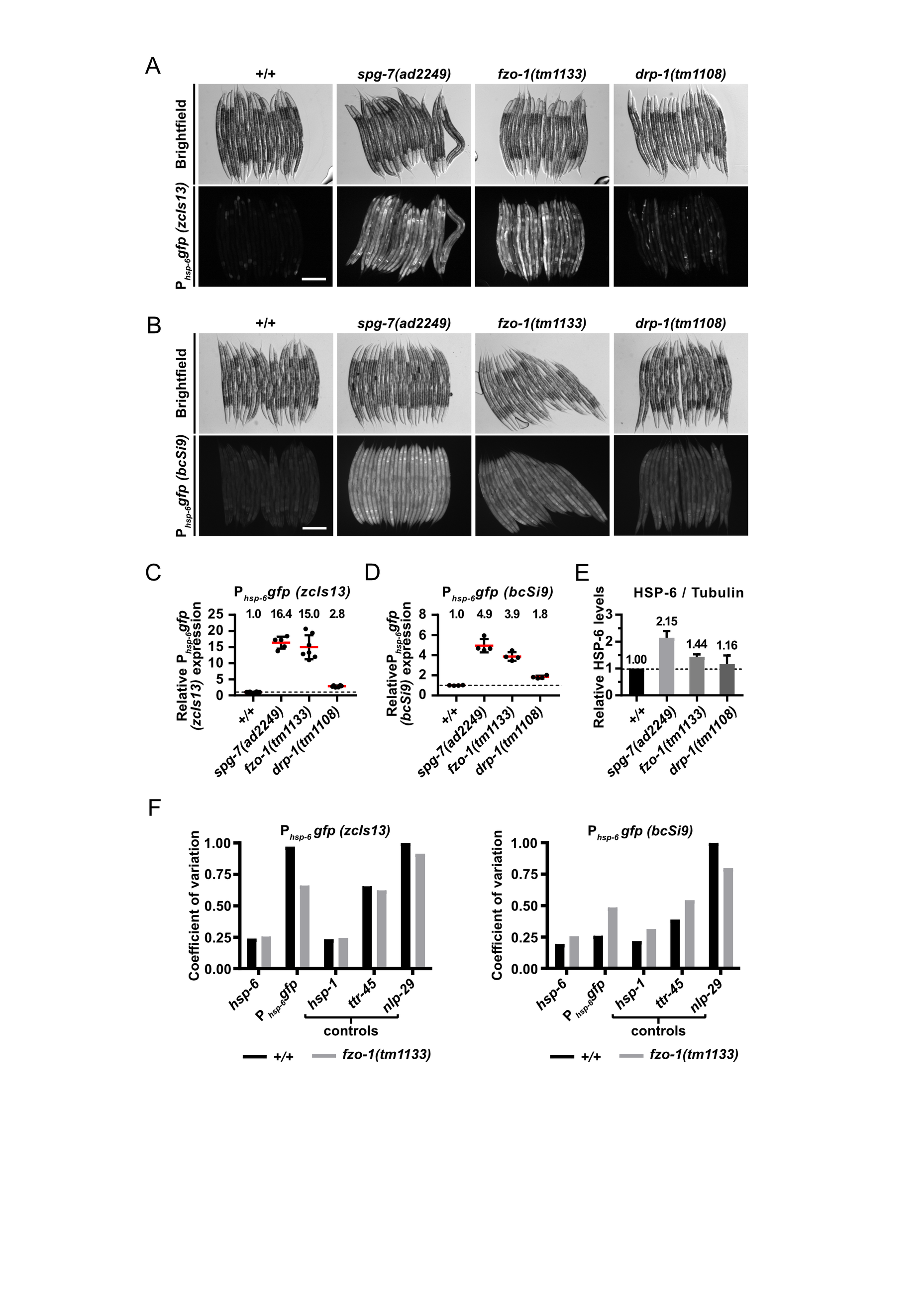

Supplement: S1 Fig — (A) Brightfield (upper panel) and fluorescence images (lower panel) of L4 larvae expressing Phsp-6gfp (zcIs13) in wild type (+/+), spg-7(ad2249), fzo-1(tm1133) or drp-1(tm1108). Scale bar: 200 μm. (B) Brightfield (upper panel) and fluorescence images (lower panel) of L4 larvae expressing Phsp-6gfp (bcSi9) in wild type (+/+), spg-7(ad2249), fzo-1(tm1133) or drp-1(tm1108). Scale bar: 200 μm. (C) Quantifications of fluorescence images of panel A (Phsp-6gfp (zcIs13)) are shown. Each dot represents quantification of 15–20 L4 larvae. Values indicate means ± SD of ≥5 independent measurements. (D) Quantifications of fluorescence images of panel B (Phsp-6gfp (bcSi9)) are shown. Each dot represents quantification of 15–20 L4 larvae. Values indicate means ± SD of ≥4 independent measurements. (E) Quantifications of western blot analysis of endogenous HSP-6 levels in wild-type (+/+), spg-7(ad2249), fzo-1(tm1133) or drp-1(tm1108) using anti-HSP-6 antibodies. For each genotype, 100 L4 larvae were harvested per experiment for western blot analysis. Values indicate means of relative HSP-6 expression (HSP-6/TUB) ± SD, n = 2. (F) Inter-individual variability in gene expression of target genes in bcSi9 and zcIs13 in both wild type (+/+) and fzo-1(tm1133). To estimate inter-individual variability in gene expression, the coefficient of variation was calculated from individual mRNA levels obtained from normalized Ct values using the delta Ct method. Inter-individual variability values were normalized such that variability values for nlp-29 in wild type = 1 (bcSi9 or zcIs13). Number of individual worms: n = 35 (bcSi9), n = 32 (bcSi9; fzo-1(tm1133)), n = 31 (zcIs13), n = 31 (zcIs13; fzo-1(tm1133)). (TIF) [file pgen.1008638.s001.tif]

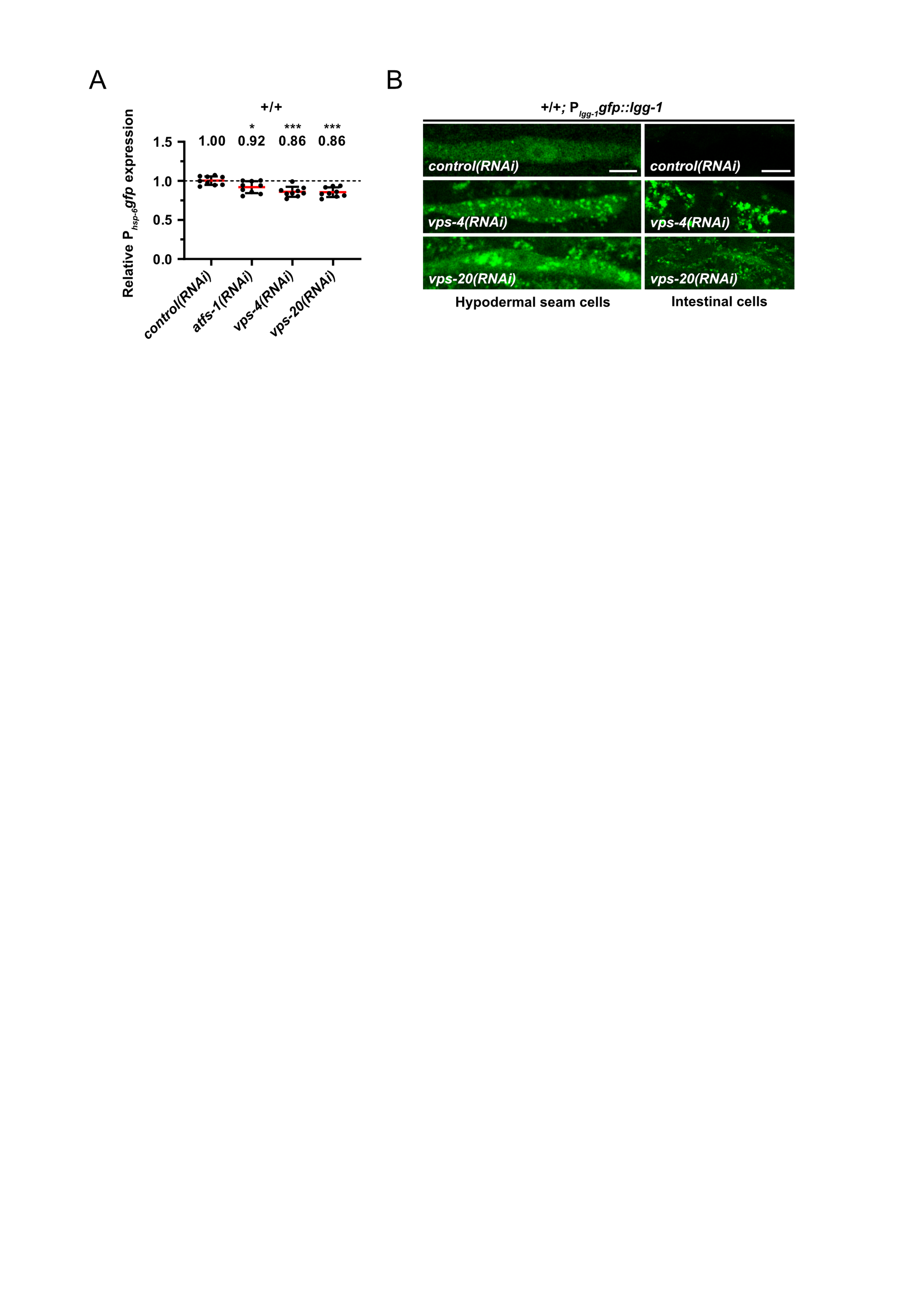

Supplement: S2 Fig — (A) L4 larvae were subjected to control(RNAi), atfs-1(RNAi), vps-4(RNAi) or vps-20(RNAi) and the F1 generation was imaged. Each dot represents the quantification of fluorescence intensity of 15–20 L4 larvae. Values indicate means ± SD of 5 independent experiments in duplicates. *P<0.05, ***P<0.001 using one-way ANOVA with Dunnett’s multiple comparison test to control(RNAi). (B) Plgg-1gfp::lgg-1 expression of L4 larvae in hypodermal seam cells and intestinal cells upon control(RNAi), vps-4(RNAi) or vps-20(RNAi). Representative images of >30 animals from two independent biological replicates are shown. Scale bar hypodermal seam cells: 5 μm. Scale bar intestinal cells: 20 μm. (TIF) [file pgen.1008638.s002.tif]

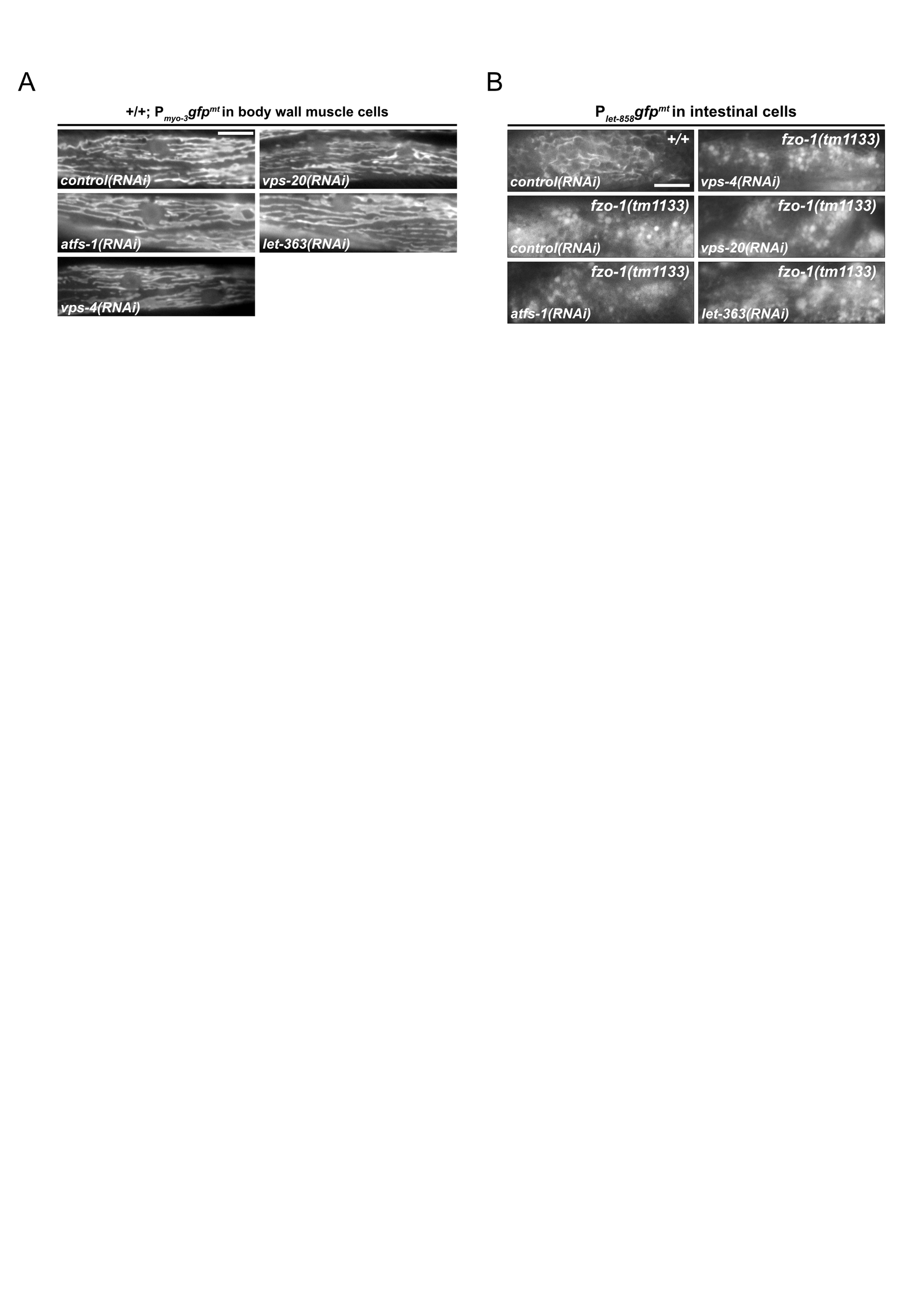

Supplement: S3 Fig — (A) Fluorescence images of L4 larvae expressing Pmyo-3gfpmt in wild type (+/+). L4 larvae were subjected to control(RNAi), atfs-1(RNAi), vps-4(RNAi), vps-20(RNAi) or let-363(RNAi) and the F1 generation was imaged. Scale bar: 10 μm. (B) Fluorescence images of L4 larvae expressing Plet-858gfpmt in wild type (+/+) or fzo-1(tm1133). L4 larvae were subjected to control(RNAi), atfs-1(RNAi), vps-4(RNAi), vps-20(RNAi) or let-363(RNAi) and the F1 generation was imaged. Scale bar: 10 μm. (TIF) [file pgen.1008638.s003.tif]

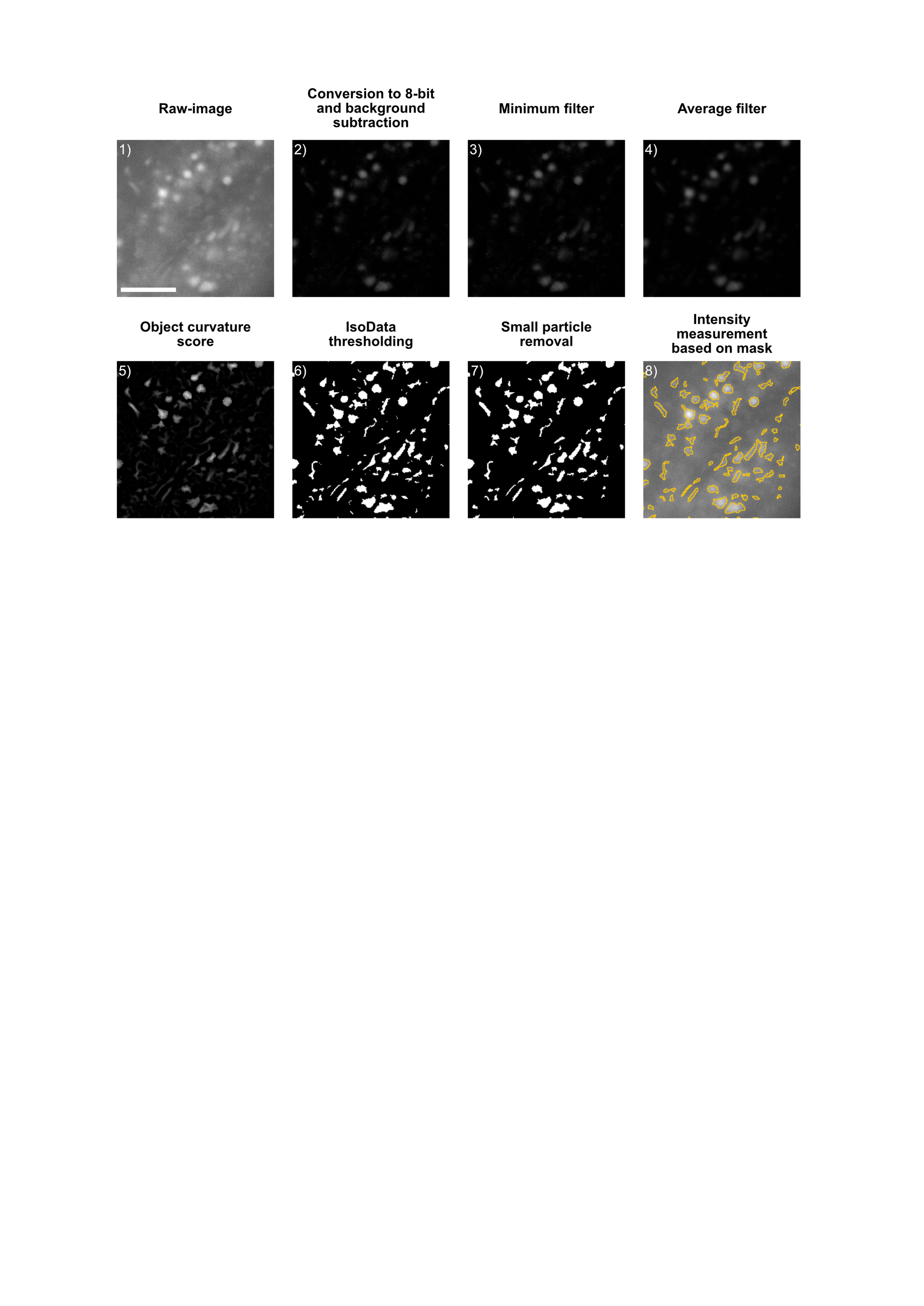

Supplement: S4 Fig — A raw 16-bit image (1) is converted to 8-bit, followed by a background subtraction using the rolling ball algorithm (2). This is followed by the successive application of a minimum (3) and average filter (4). The ImageJ Tubeness plugin generates an image with object curvature scores (5), after which the IsoData autothresholding is applied to generate the binary mask (6). Noise is removed by filtering out particles below a certain size (7) and the final mask is used to define the area in which intensity measurements are obtained (8). Scale bar: 5 μm. (TIF) [file pgen.1008638.s004.tif]

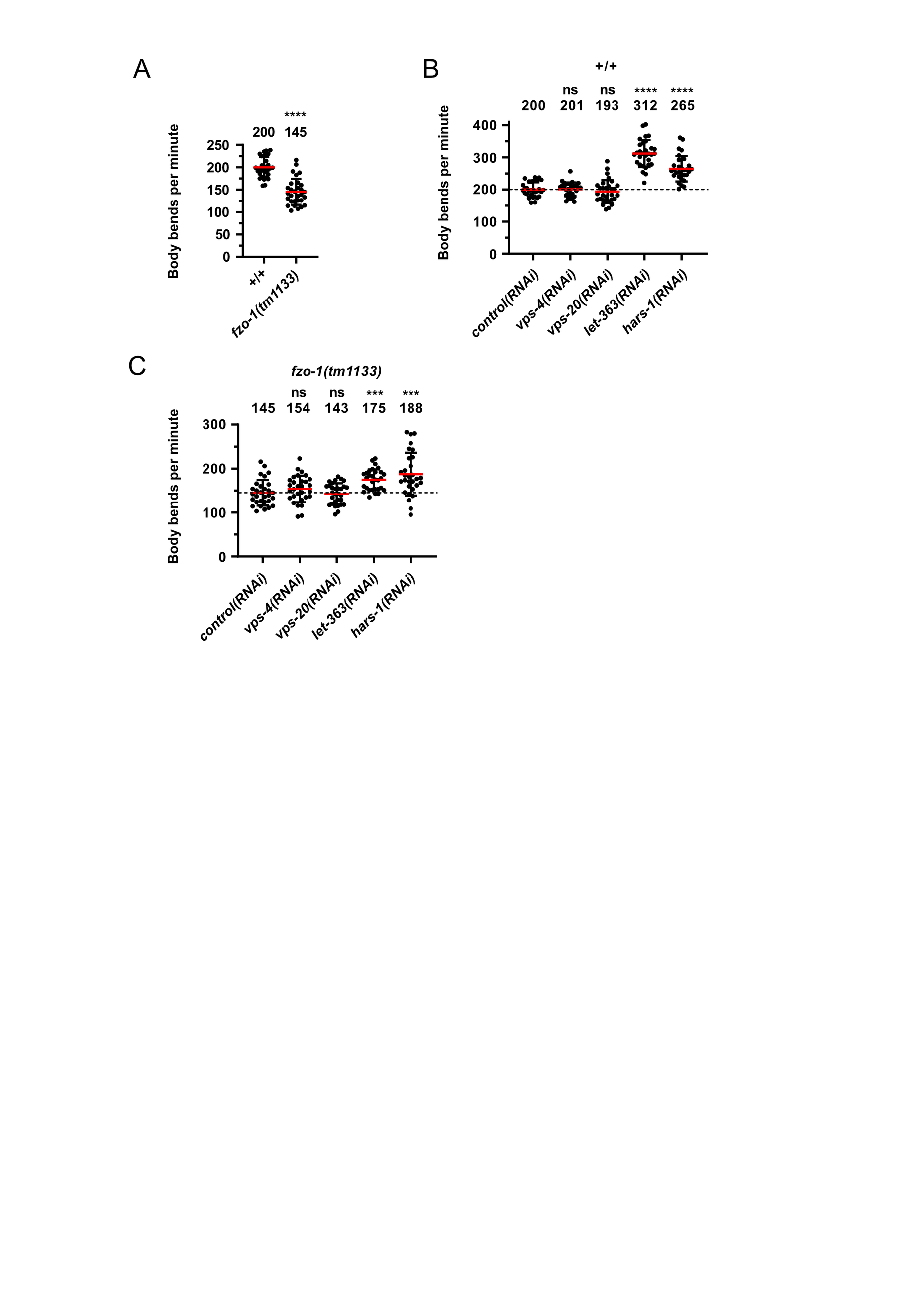

Supplement: S5 Fig — Thrashing rate was analyzed by counting body bends of animals swimming for 1 minute in M9 buffer in 3 independent experiments. Each dot represents one L4 larvae. (A) Thrashing rates of wild-type (+/+) or fzo-1(tm1133) L4 larvae. ****P<0.0001 using unpaired two-tailed t-test. n = 30. (B) Thrashing rates in wild-type animals upon induction of autophagy. L4 larvae were subjected to control(RNAi), vps-4(RNAi), vps-20(RNAi), let-363(RNAi) or hars-1(RNAi) and the F1 generation was analyzed. ns: not significant, ****P<0.0001 using Kruskal-Wallis test with Dunn’s multiple comparison test to control(RNAi). n = 30. (C) Thrashing rates in fzo-1(tm1133) animals upon induction of autophagy. L4 larvae were subjected to control(RNAi), vps-4(RNAi), vps-20(RNAi), let-363(RNAi) or hars-1(RNAi) and the F1 generation was analyzed. ns: not significant, ***P<0.001 using Kruskal-Wallis test with Dunn’s multiple comparison test to control(RNAi). n = 30. (TIF) [file pgen.1008638.s005.tif]

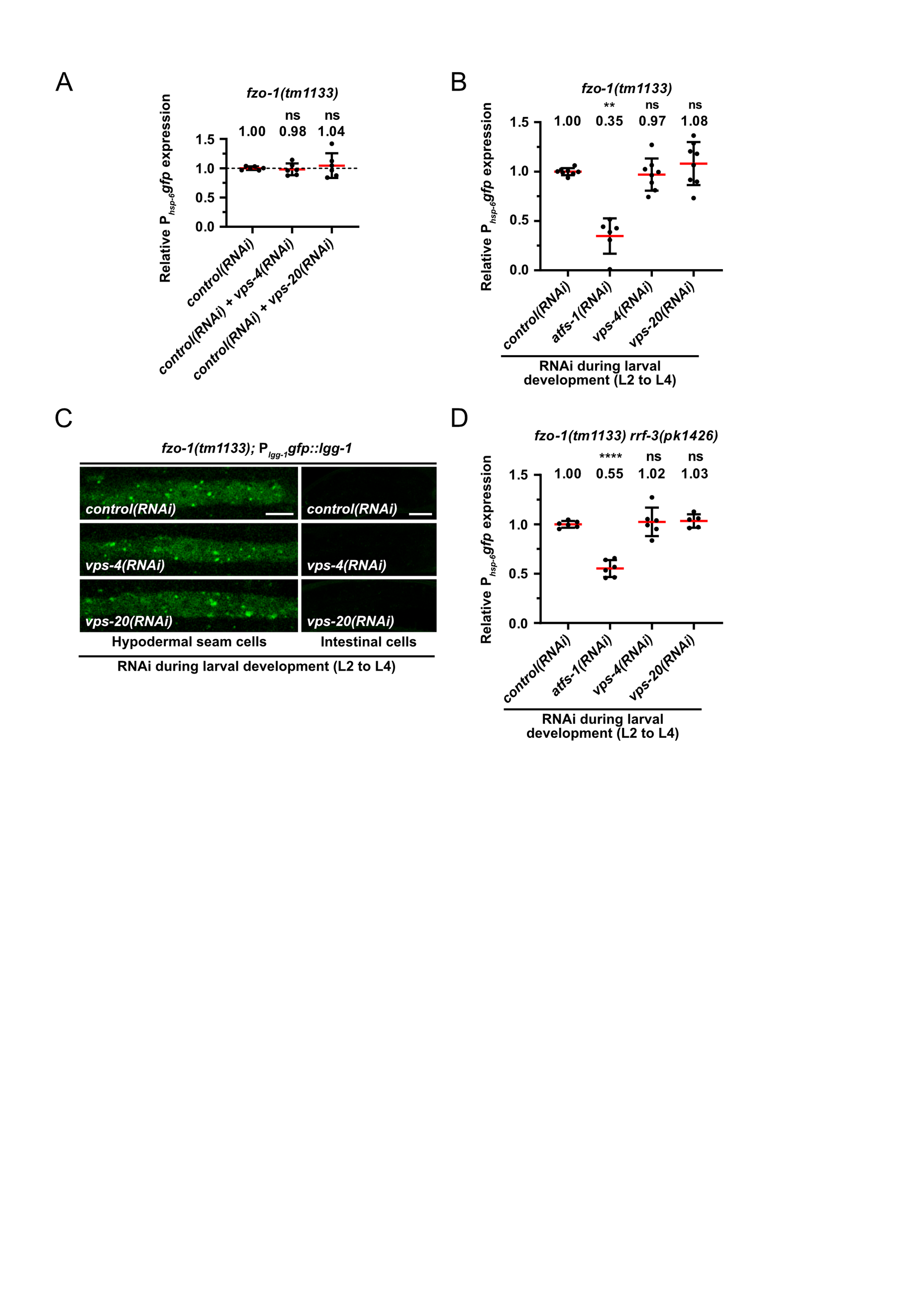

Supplement: S6 Fig — (A) Quantifications of fluorescence images of L4 larvae expressing Phsp-6gfp (bcSi9) in fzo-1(tm1133). Each ESCRT(RNAi) was diluted 1:1 with control(RNAi). After subtracting the mean fluorescence intensity of wild type (+/+) on control(RNAi), the values were normalized to fzo-1(tm1133) on control(RNAi). Each dot represents the quantification of fluorescence intensity of 15–20 L4 larvae. Values indicate means ± SD of 3 independent experiments in duplicates. ns: not significant, using one-way ANOVA with Dunnett’s multiple comparison test to control(RNAi). (B) Quantifications of fluorescence images of L4 larvae expressing Phsp-6gfp (bcSi9) in fzo-1(tm1133). L2 larvae were subjected to control(RNAi), atfs-1(RNAi), vps-4(RNAi) or vps-20(RNAi) and the same animals were imaged in L4 stage. After subtracting the mean fluorescence intensity of wild type (+/+) on control(RNAi), the values were normalized to fzo-1(tm1133) on control(RNAi). Each dot represents the quantification of fluorescence intensity of 15–20 L4 larvae. Values indicate means ± SD of 4 independent experiments in duplicates. ns: not significant, **P<0.01 using Kruskal-Wallis test with Dunn’s multiple comparison test to control(RNAi). (C) Plgg-1gfp::lgg-1 expression of fzo-1(tm1133) L4 larvae in hypodermal seam cells and intestinal cells. L2 larvae were subjected to control(RNAi), vps-4(RNAi) or vps-20(RNAi) and the same animals were imaged in L4 stage. Representative images of >60 animals from two independent biological replicates are shown. Scale bar hypodermal seam cells: 5 μm. Scale bar intestinal cells: 20 μm. (D) Quantifications of fluorescence images of L4 larvae expressing Phsp-6gfp (bcSi9) in fzo-1(tm1133) rrf-3(pk1426). L2 larvae were subjected to control(RNAi), atfs-1(RNAi), vps-4(RNAi) or vps-20(RNAi) and the same animals were imaged in L4 stage. After subtracting the mean fluorescence intensity of wild type (+/+) on control(RNAi), the values were normalized to fzo-1(tm1133) on control(RNAi). Each [file pgen.1008638.s006.tif]

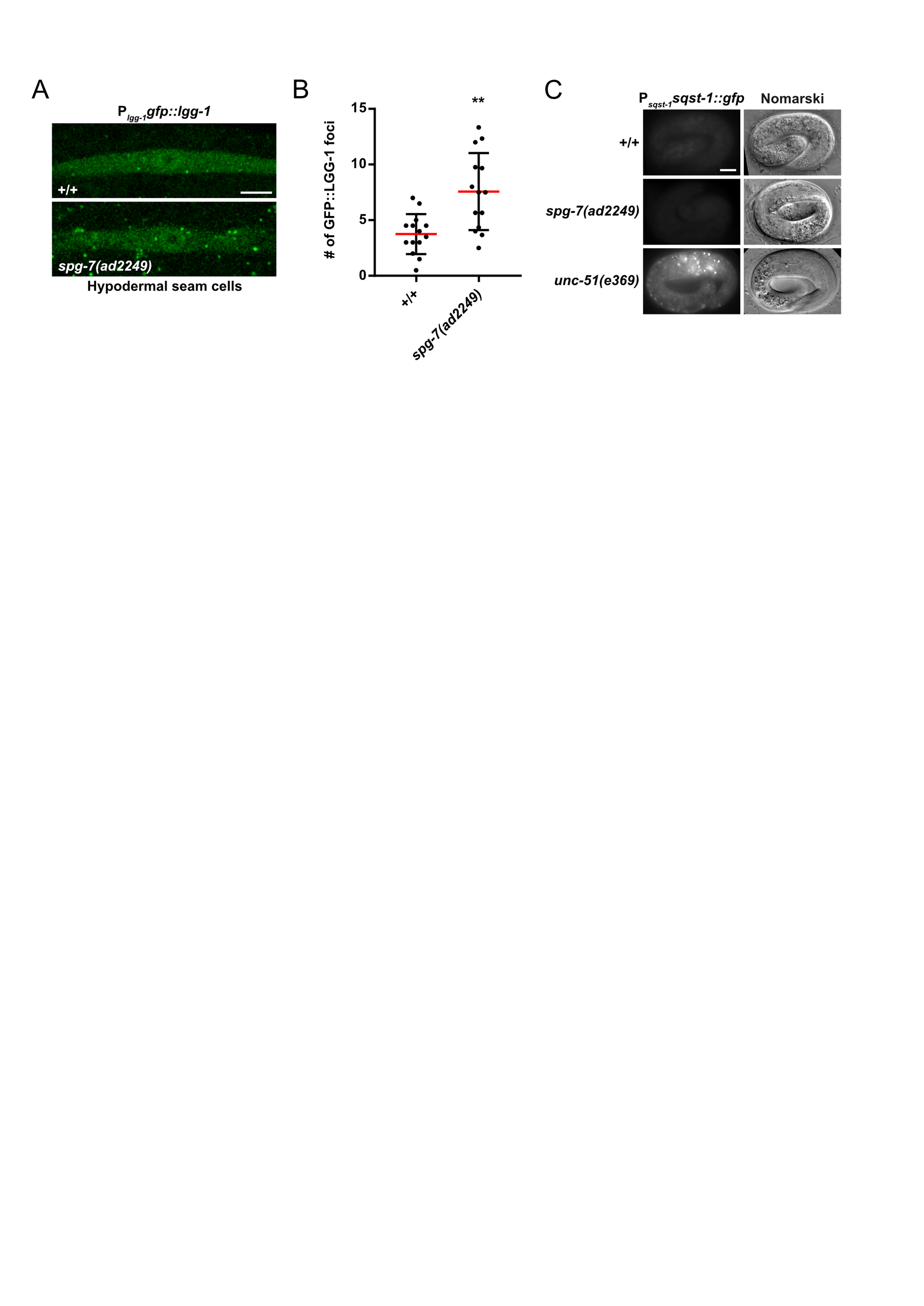

Supplement: S7 Fig — (A) Plgg-1gfp::lgg-1 expression in hypodermal seam cells of wild type (+/+) or spg-7(ad2249) L4 larvae. Scale bar: 5 μm. (B) Quantification of GFP::LGG-1 foci in hypodermal seam cells from panel A. Each dot represents the average amount of GFP::LGG-1 foci counted from 2–5 seam cells in one animal. n≥18 for each genotype; values indicate means ± SD; **P<0.01 using unpaired two-tailed t-test with Welch’s correction. (C) Nomarski and fluorescent images of the Psqst-1sqst-1::gfp translational reporter in embryos of wild type (+/+) and spg-7(ad2249) animals. As a positive control for a block in autophagy, unc-51(e369) was used. Representative images of >60 embryos are shown. Scale bar: 10 μm. (TIF) [file pgen.1008638.s007.tif]

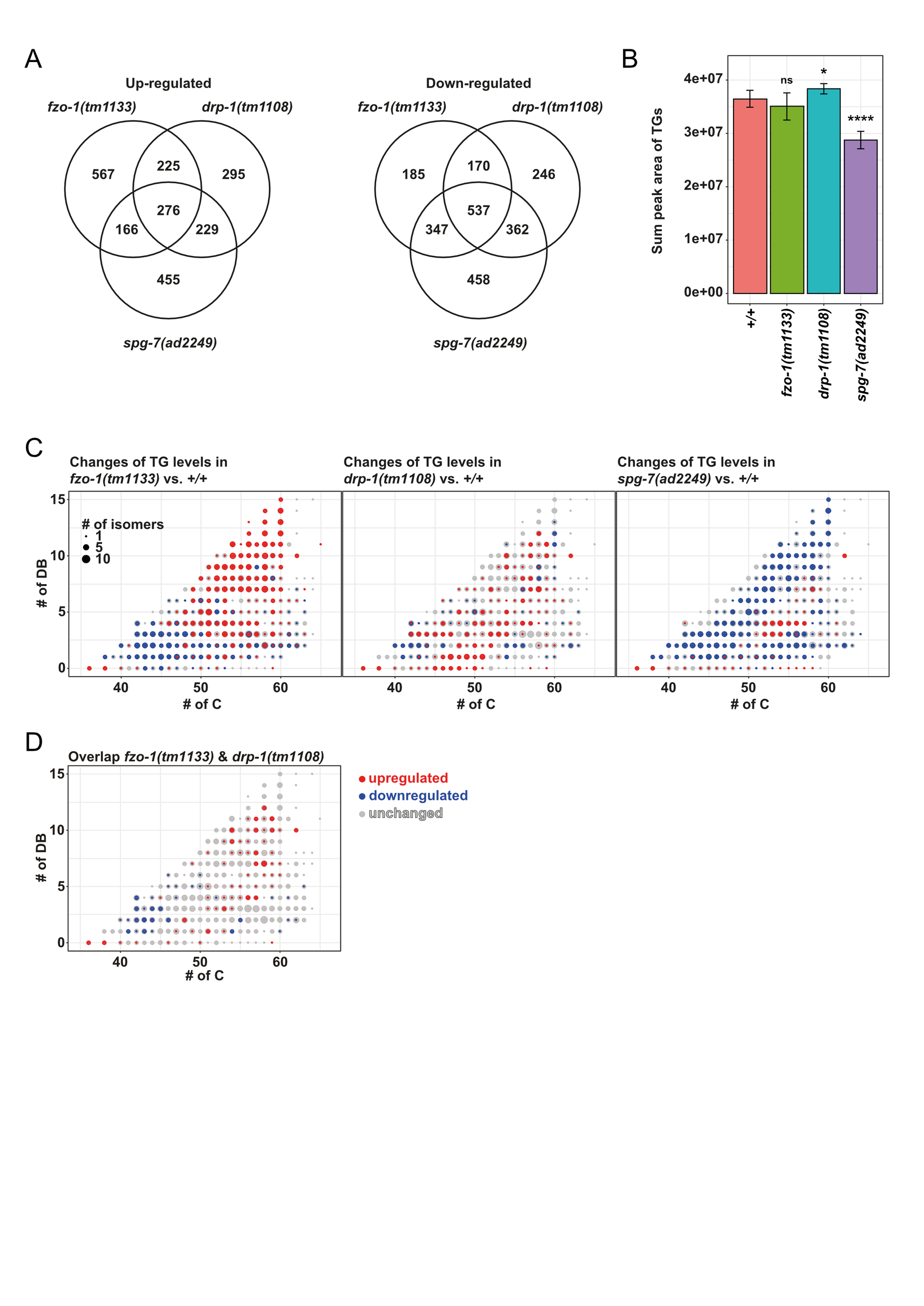

Supplement: S8 Fig — (A) Venn diagrams showing the overlap of lipids up- or downregulated in fzo-1(tm1133), drp-1(tm1108) and spg-7(ad2249) in comparison to wild type (+/+). (B) Total amount of TGs in wild type (+/+), fzo-1(tm1133), drp-1(tm1108) and spg-7(ad2249) backgrounds. Means ± SD are shown; ns: not significant, *P<0.05, ****P<0.0001 using Welch test. (C) Scatterplot indicating the distribution and changes in the levels of TG species in the different mutants in comparison to wild type (+/+). (D)) Scatterplot indicating the overlap of the changes in the levels of TG species of fzo-1(tm1133) and drp-1(tm1108) mutants in comparison to wild type (+/+). (C) and (D) The x-axis labels the number of carbons (# of C) and the y-axis the number of double bonds (DB) in the acyl sidechains. The size of a dot indicates the number of detected isomers for a specific sum composition. Grey dots represent all detected TGs species and blue and red dots indicate down- (blue) or upregulation (red). (TIF) [file pgen.1008638.s008.tif]

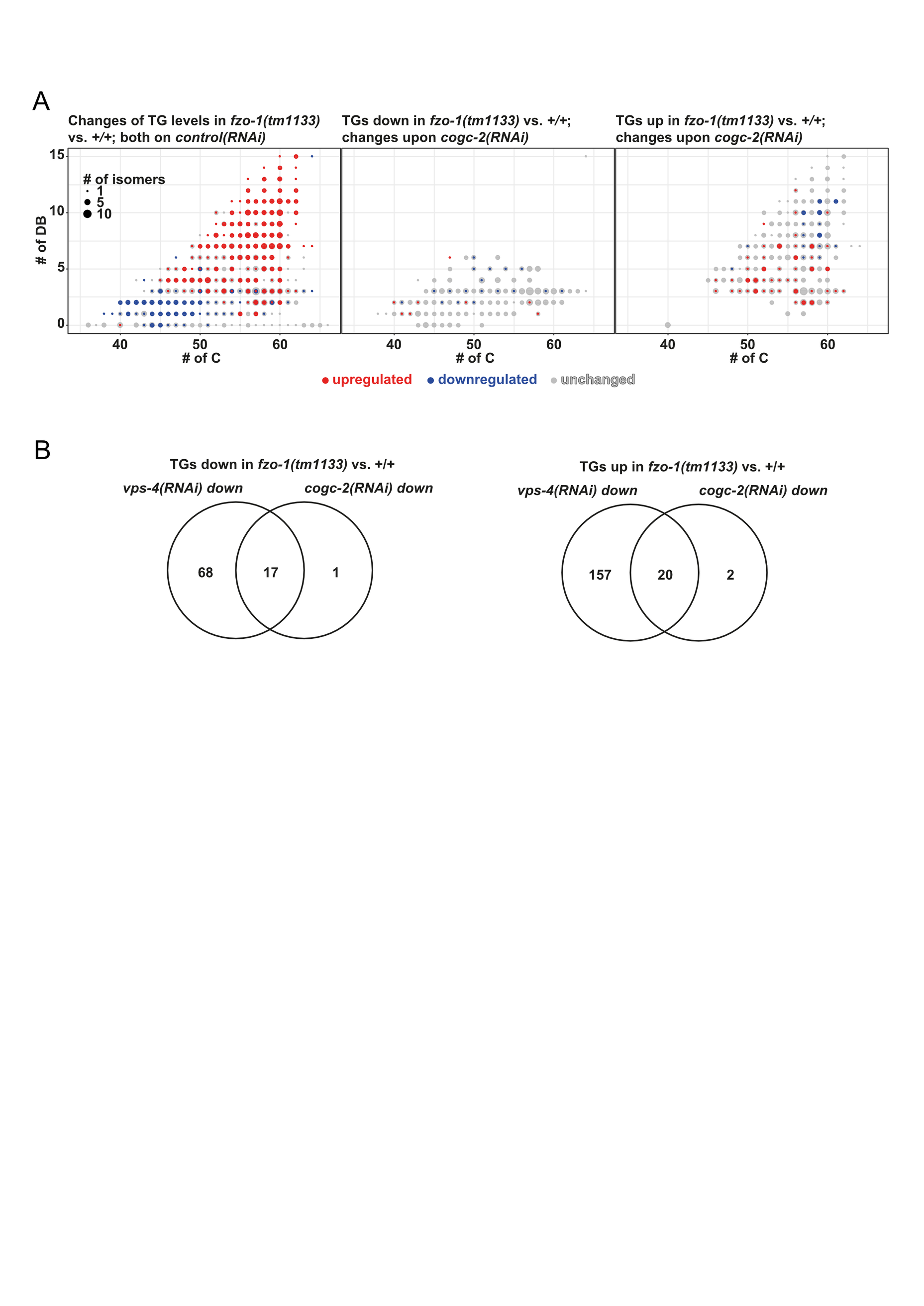

Supplement: S9 Fig — (A) Scatterplot indicating the distribution and changes in the level of TG species in fzo-1(tm1133) mutants in comparison to wild type (+/+). The x-axis labels the number of carbons (# of C) and the y-axis the number of double bonds (DB) in the acyl sidechains. The size of a dot indicates the number of detected isomers for a specific sum composition. Grey dots represent all detected TGs species and blue and red dots indicate down- (blue) or upregulation (red). (B) Venn diagram indicating the overlap of TG species downregulated (left panel) or upregulated (right panel) in fzo-1(tm1133) and downregulated upon vps-4(RNAi) or cogc-2(RNAi). (TIF) [file pgen.1008638.s009.tif]
