# Supplementary material for: Autophagy compensates for defects in mitochondrial dynamics
Source: PLoS Genet. 2020 Mar 19;16(3):e1008638. doi: 10.1371/journal.pgen.1008638 (PMC7135339; doi:10.1371/journal.pgen.1008638)
Supplement: Attachment — Submitted filename: Rebuttal letter_22December2019.pdf [file pgen.1008638.s014.pdf]

Reviewer #1: This manuscript describes a relatively unexplored relationship between autophagic flux and the regulation of the UPRmt caused by impaired mitochondrial dynamics. A genome-wide RNAi screening approach was used to identify genes that are required for the activation of the UPRmt when mitochondrial fusion is compromised. 299 genetic suppressors of the fzo-1(tm1133) mutant-mediated activation of the UPRmt were identified from this screen of which three were components of the ESCRT. Interestingly, knockdown of these components do not rescue the defects in mitochondrial morphology observed with fzo-1(tm1133) but instead increase mitochondrial membrane potential which is believed to be related to the suppression of the UPRmt observed in this mutant. Using multiple assays, it was shown that RNAi against the ESCRT genes increases autophagy flux in wild-type animals and even more so in the fzo-1(tm1133) mutant. The connection between autophagic flux and the suppression of fzo-1(tm1133) UPRmt activation was validated with knockdown of let-363/mTOR which stimulates autophagy. Remarkably, blocking mitophagy using a pdr-1 mutant did not prevent the suppression of the UPRmt by fzo-1(tm1133). The authors further expanded their findings by testing the remainder of suppressors that had been identified for changes in autophagic flux, of which RNAi of 126 genes increased autophagy (as well as 17 with known autophagy roles totaling 143 suppressors). Finally, while most of the 143 genes also suppressed drp-1(tm1108) induction of the UPRmt, 90 also suppressed the UPRmt activation unrelated to the regulation of mitochondrial dynamics (i.e. the spg-7(ad2249 mutant). Overall the manuscript is well written and explores an interesting correlation between the regulation of the UPRmt with impaired mitochondrial dynamics and autophagic flux, albeit with little mechanism.

1) One of the main interpretations of this manuscript is that increased autophagic flux suppresses the UPRmt resulting from impaired mitochondrial dynamics by restoring mitochondrial homeostasis. However, this conclusion is based primarily on the increase in mitochondrial membrane potential observed with TMRE staining. Are other parameters in mitochondrial function improved as well such as oxygen consumption and ATP production?

Haeussler et al: *The reviewer raises an important point, which we tried to address using various approaches. As outlined below, unfortunately, for technical reasons, we were unable to determine whether other parameters are improved as well. The variable, pleiotropic effects caused by the knock-down of the relevant genes may have contributed to this.*

In collaboration with Andrew Wojtovich (University of Rochester Medical Center), we planned to measure mitochondrial function using a Clark oxygen electrode. However, these experiments require large amounts of staged worms (~ 0.5 million worms), because they need to be done with isolated mitochondria. Unfortunately, we were unable to obtain sufficient amounts of staged fzo-1(tm1133) animals due to their variable developmental delays.

In collaboration with Fabiana Perocchi (Helmholtz Centre Neuherberg, Germany), we tried to use the Seahorse Analyzer XF96 to test if induction of autophagy by knockdown of ESCRT components has an effect on maximal respiratory capacity since it was previously shown that fzo-1(tm1133) animals have a lower maximal respiratory capacity, while basal OCR was found to be unchanged (Luz et al., 2015). Unfortunately, uncoupling of OXPHOS did not reproducibly occur after addition of FCCP. Initially, we tested different FCCP concentrations in order to uncouple OXPHOS but did not see a response for wild-type or fzo-1(tm1133) animals using M9

buffer containing PEG to prevent worms from sticking to plastic (see ***Figure R1****). Previously, concentrations of 10 µM and 15 µM were used ((Koopman et al., 2016; Luz et al., 2015). Furthermore, we tested the basal OCR upon knock-down of ESCRT components (see* ***Figure R2****). The high variability of OCR between the eight technical replicates of each condition did not allow any conclusions. Again, FCCP injection did not result in uncoupling of mitochondria. After these attempts, we contacted the two labs that have published Seahorse Analyzer experiments with C. elegans (Meyer lab at Duke University and Houtkooper lab at Laboratory Genetic Metabolic Diseases, Academic Medical Center, Amsterdam) and we changed our setup as suggested by them. Both labs suggested using different buffers, which we tried (M9 without PEG and unbuffered "EPA water"). Additionally, we varied the number of worms in both conditions, using 10 L4 larvae/well and 25 L4 larvae/well (see* ***Figure R3****). Using M9 buffer, we could see uncoupling of OXPHOS in fzo-1(tm1133) upon injection of 15 µM FCCP in the wells containing 25 L4 larvae. Following this experiment, we knocked-down ESCRT components in wild-type and fzo-1(tm1133) animals in order to measure OCR after induction of autophagy (see* ***Figures R4&R5****). Although using the exact same conditions, we could, unfortunately, not reproduce the uncoupling of mitochondria with FCCP. Thus, we discontinued these experiments since we could not reproducibly establish a reliable protocol.*

We also attempted to measure total ATP levels in fzo-1(tm1133) animals with and without the induction of autophagy. Johnson and Nehrke published that the AMP/ATP ratio was unchanged in fzo-1(tm1133) mutants when compared to wild type (Johnson and Nehrke, 2010). We measured total ATP levels in three independent experiments using a previously published luciferase assay (Palikaras and Tavernarakis, 2016) and found that the results were highly variable among the three replicates and therefore not conclusive (see ***Figures R6&R7****).*

2) Related to 1): are there any physiological differences in fzo-1(tm1133) animals when autophagic flux is increased that might support the author's claim of improved cellular homeostasis? For example, thrashing rate, developmental rate, or possibly lifespan?

Haeussler et al: *As suggested by the reviewer, we tested whether the induction of autophagy in fzo-1 mutants through knock-down of let-363, hars-1, vps-4 or vps-20 has an effect at the organismal level.*

First, we tested life span. It has previously been shown that attenuation of translation or depletion of let-363 extends life span in C. elegans (Hansen et al., 2007; Kennedy and Kaeberlein, 2009; Vellai et al., 2003). Unfortunately, we were unable to observe life span extension for any of the genes tested (let-363, hars-1, vps-4, vps-20) (see ***Figure R8****). However, we did observe decreased maximal life span upon knock-down of the two ESCRT genes vps-4 and vps-20 in both wild type and fzo-1 mutants. As mentioned above, knock down of ESCRT genes can cause embryonic and larval lethality (Djeddi et al., 2012). The observed reduction in maximal life span therefore most likely is a consequence of these developmental defects. Furthermore, even though the induction of autophagy is widely believed to correlate with extension of life span, Zhou and colleagues recently showed that the induction of autophagy per se does not predict life span (Zhou et al., 2019).*

Second, we analyzed trashing rates as a proxy for worm fitness since Johnson and Nehrke found that trashing rate is decreased in fzo-1(tm1133) (Johnson and Nehrke, 2010). We found that thrashing rates were increased for let-363(RNAi) and hars-1(RNAi) in fzo-1(tm1133) and wild type while vps-4(RNAi) and vps-20(RNAi) did not show any changes in thrashing rates in either background (see ***Figure S5****). This data is now included in the Result section of the revised manuscript on* ***page 16*** *and we discuss these results on* ***page 24*** *of the Discussion section.*

3) The authors also connect the increase in membrane potential with the regulation of ATFS-1 which is based on mitochondrial import efficiency. Is mitochondrial import improved in fzo-1(tm1133) animals with increased autophagic flux? It would be interesting to examine import efficiency using mitochondrial targeted GFP similar to what was described in Nargund et al. 2012.

Haeussler et al: *The reviewer addresses an important point. Indeed, earlier this year, Rolland and colleagues showed that the mitochondrial import efficiency of ATFS-1 directly determines whether UPR^{mt} is induced or not (Rolland et al., 2019). We found that upon autophagy induction, TMRE signal intensity and, hence, mitochondrial membrane potential is increased in fzo-1(lf) animals. Since increased mitochondrial membrane potential increases mitochondrial import efficiency, in line with the Rolland et al study, we conclude that this leads to enhanced mitochondrial import of ATFS-1 and, consequently, the suppression of fzo-1(lf)-induced UPR^{mt}. For this reason, we did not attempt to establish mitochondrial import assays, which we also believe are beyond the scope of this study.*

4) The authors were not able to show that increased autophagy is necessary for the suppression of the UPRmt in fzo-1(tm1133) animals following ESCRT RNAi because of the embryonic lethality that is observed when the RNAi is performed in the fzo-1(tm1133; unc-51(e369) double mutant background. This is unfortunate since this experiment is rather important to test their central hypothesis. Would a milder form of unc-51 knockdown prevent this associated lethality? Possibly double RNAi against unc-51 and vps-4/20/27 in the fzo-1(tm1133) background? Or introducing a RNAi-hypersensitive mutant (e.g. rrf-3) into the fzo-1(tm1133); unc-51(e69) background which might allow for RNAi to occur in larvae?

Haeussler et al: *We thank the reviewer for these suggestions. As suggested, we tested if diluted ESCRT(RNAi) can suppress fzo-1(lf)-induced UPR^{mt} and found that dilution of vps-4(RNAi) and vps-20(RNAi) with control(RNAi) was not able to suppress (see* ***Figure S6A****). Furthermore, we crossed the RNAi-sensitizing mutation lin-35(n745) into fzo-1(tm1133); bcSi9 mutants but found that the resulting strain was too sick to be used in RNAi-experiments. We therefore crossed another RNAi-sensitizing mutation, rrf-3(pk1426), into fzo-1(tm1133); bcSi9 and knocked-down vps-4 and vps-20 during larval development. However, again, we could not detect a decrease in fzo-1(lf)-induced UPR^{mt} in these experiments (see* ***Figure S6D****). This data is now included in the Result section of the revised manuscript on* ***page 16&17****.*

5) on p.13, let-363 RNAi suppresses the UPRmt reporters in fzo-1(tm1133) animals to approximately the same level as their control ges-1::GFP. The authors then state however that let-363 RNAi increases TMRE by 19%, supporting their claim that autophagic flux suppresses this UPRmt activation by restoring mitochondrial membrane potential. However, the 19%

increase does not appear to be significant based on their quantification in Figure 2F. Is this statement valid then? The same issue also applies to their finding that hars-1 RNAi suppresses ges-1::GFP expression and leads to a 12% increase in TMRE fluorescence which, according to their graph in 2F, is not significant.

Haeussler et al: *To address the reviewer's comments, we increased the number of repeats for TMRE stainings upon hars-1(RNAi) and let-363(RNAi). let-363(RNAi) leads to increased TMRE staining intensity by 14% and hars-1(RNAi) by 31% (see* ***Figure 2F****). Upon hars-1(RNAi) we now also see a statistically significant increase in TMRE intensity and therefore mitochondrial membrane potential. let-363(RNAi) on the other hand still does not show a statistically significant increase in TMRE stainings. However, looking at the graphs carefully, it is clear that depletion of let-363 in fzo-1(tm1133) does have an effect on TMRE staining intensity. In about half of the animals, an increase in TMRE staining intensity is detectable. The other half seems to have decreased TMRE staining intensity. This may be due to variant efficiency of RNAi. This data is included in the Result section of the revised manuscript on* ***page 15****.*

Reviewer #2: This is a solid piece of work describing an unexpected signaling axis between regulators of mitochondrial dynamics, the mitochondrial unfolded protein response, and autophagy. The approach is generally sophisticated, rigorous, well-executed, and appropriate for PLoS Genetics, and the results will contribute significantly to the field. I want to be clear that I am very enthusiastic regarding this story. I used this manuscript to train one of my postdoctoral fellows in the review process, which contributed to its length. Within that context, I hope that the criticisms detailed below will help to make the manuscript even stronger. I have listed a few major points, the first of which should be addressed experimentally, followed by minor points and suggestions to improve clarity and flow, which may be addressed at the author's discretion but should not require additional experiments.

Major points:

1. Line 240. The TMRE result needs to be interpreted with caution. Obviously, there is much less staining in fzo-1(tm1133) mutants, consistent with fragmentation having been associated previously with loss of MMP. However, while ESCRT(RNAi) suppresses this loss, the fact that suppression is modest at best and is extremely variable warrants careful consideration of TMRE's dye loading, distribution, and equilibrium, which may not be identical in worms of differing genotypes. Moreover, mitochondrial content may differ, as well – which may be quite relevant, as atfs-1 dependent mitoUPR induction may drive a mitogenesis program upon fragmentation. In addition, TMRE can be used in both quenching and nonquenching modes, which depends upon its concentration and causes changes in mito membrane potential to present oppositely. To specifically address these issues, I would suggest querying mito::nuclear DNA ratios using qPCR, applying MitoTracker Green as a non-MMP dependent assessment of mito content, ascertaining that you are working in non-quench mode, and employing a separate measure of membrane potential to confirm the TMRE result for several of the most relevant target genes. These techniques in parallel will enhance scientific rigor and greatly strengthen the conclusion.

Haeussler et al: *We thank the reviewer for this comment, because it made us realize that we had not explained well enough how we quantify the TMRE signal. The 'TMRE fluorescence intensity per mitochondrial area' was measured by segmentation of mitochondria in the recorded images, followed by quantification of fluorescence intensity only in the segmented areas i.e. the mitochondria. A schematic describing the workflow is now presented in* ***Figure S4*** *and the Result section was modified accordingly on* ***page 11****. Furthermore, the TMRE concentrations we used are non-quenching and therefore suitable for quantifications and direct correlations with mitochondrial membrane potential. The revised manuscript was updated in the Methods section on* ***page 33****.*

2. Line 307. The claim that let-363(RNAi) increased mito membrane potential (MMP) in the fzo-1(tm1133) mutant is not supported by the data. Even though there's a 19% increase in signal, it is not statistically significant. This may be concerning, since let-363 is marketed as being a "control" regulator that ties together autophagy and MMP.

Haeussler et al: *To address the reviewer's comments, we increased the number of repeats for TMRE stainings upon hars-1(RNAi) and let-363(RNAi). let-363(RNAi) leads to increased TMRE staining intensity by 14% and hars-1(RNAi) by 31% (see* ***Figure 2F****). Upon hars-1(RNAi) we now also see a statistically significant increase in TMRE intensity and thereby mitochondrial membrane potential. let-363(RNAi) on the other hand still does not show a statistically significant increase in TMRE stainings. However, looking at the graphs carefully, it is clear that depletion of let-363 in fzo-1(tm1133) does have an effect on TMRE staining intensity. In about half of the animals, an increase in TMRE staining intensity is detectable. The other half seems to have decreased TMRE staining intensity. This may be due to variant efficiency of RNAi. This data is included in the Result section of the revised manuscript on* ***page 15****.*

3. Line 413. The logic of saying that the hars-1(RNAi) phenotype supports a role for genes in the GO-terms "Translation" and "Ribosomal Biogenesis" having a beneficial effect on MMP is faulty. Not only in the hars-1(RNAi) effect on MMP not statistically significant, there is no evidence to support this conclusion except that hars-1 also falls under these GO-terms. This conclusion should be removed or undergo a severe reduction in strength to reflect the very weak support.

Haeussler et al: *We agree with the reviewer and addressed this comment by changing the wording in the manuscript. In addition, as stated above, we increased the number of worms quantified in TMRE stainings which results in higher fold change that is also statistically significant (see* ***Figure 2F****). This data is included in the result section of the revised manuscript on* ***page 15****.*

Minor points:

4. Line 83. Strictly speaking, ATFS-1 does not translocate to the nucleus (from the mito), even though often referred to in the context of retrograde mito-nuclear signal transduction. For clarity, I would encourage "targeting of ATFS-1 is redirected from the mito to the nucleus" or some variant thereof. This comment applies as well to similar statements in the discussion.

Haeussler et al: *We agree with the reviewer and have changed the wording in the Introduction section on* ***page 4****.*

Lines 155-186. This entire section is somewhat distracting. It is not intuitive as to why the multicopy transgene would be better for screening, particularly since the MosSCI transgene is used for screening later in the paper, as well as all of the validation approaches. Regardless, the authors may want to consider holding off on introducing the MosSCI allele until they actually use it. Truth-be-told, the Results section could begin with line 188 without losing any impact, and I think this would make for a tighter story.

Haeussler et al: *We thank the reviewer for this comment, which we tried to address by making the suggested changes to the text. However, at the end, we felt that the overall flow of the paper was less disrupted by presenting the data on both transgenes in the beginning of the Results section rather than in different parts. For this reason, we decided to return to the order as it was in the original manuscript.*

5. General comment. Please double-check that you are consistent in the use of past versus present tense.

Haeussler et al: *We have now double-checked and fixed this.*

6. Line 220. It is likely worth reiterating here for readers outside the field that hsp-60 is an alternative atfs-1 dependent mitoUPR target gene. It is also worth considering that hsp-6 and hsp-60, while canonical readouts for the mitoUPR, may not accurately reflect all aspects of its induction (take for example the difference between the atfs-1 gf mutant and spg-7 RNAi, even though they both induce the hsps).

Haeussler et al: *Thank you for pointing this out. In the revised manuscript, we introduce hsp-60 as an alternative UPRmt target on* ***lines 199-202*** *on* ***page 9*** *in the Introduction section. And we agree with the reviewer with regards to the canonical readouts (hsp-6 and hsp-60 transgenes) used in the UPRmt field. Indeed, that is why we also measured the level of HSP-6 and HSP-60 protein as well as mitochondrial membrane potential.*

Figure 2F. The data shows that vps RNAI partly suppresses the fzo-1 lf mutant effect on MMP. Do any of these RNAi treatments have an effect on MMP in a wildtype background?

Haeussler et al: *To address this comments, we tested RNAi against these genes also in a wild-type background and found that TMRE fluorescence intensity was, in contrast to the fzo-1(tm1133) background, decreased (see revised* ***Figures 2G&2H****). This data is described in the Result section of the revised manuscript on* ***pages 11 and 15****.*

7. Figure 3D, E. I think that it would be more appropriate to assess the ratio of cleaved GFP to GFP::LC3 rather than to tubulin. Is there a good argument for using tubulin?

Haeussler et al: *We think that tubulin is more appropriate since there are two things to consider in this context. First, there may or may not be upregulation of steady-state GFP::LGG-1 upon autophagy induction, depending on the gene that is being knocked-down and the efficiency of the RNAi. Second, GFP::LGG-1 turnover directly correlates with autophagic flux. For example, see* ***Figure 4C****; smgl-1(RNAi) leads to a drastic increase in GFP::LGG-1 and*

simultaneously to increased levels of 'cleaved GFP'. If normalized to GFP::LGG-1, this would result in a ratio slightly above 1 which would suggest that there is no induction of autophagy. For this reason, we decided to rely on the housekeeping gene tubulin for normalization.

8. Why is there no quantitation of the LGG foci in Figure 3E?

Haeussler et al: *We did not quantify the images in Figure 3E for two reasons: first, quantification of GFP::LGG-1 dots is close to impossible in the intestine and also in hypodermal seam cells (see images); second, we already quantify autophagic flux in whole animal extracts by measuring steady state levels of GFP::LGG-1 and 'cleaved GFP' using western blots.*

9. Did you test whether unc-51 suppresses the effect of vps RNAi in the fzo-1 lf mutant? If so, it is worth mentioning.

Haeussler et al: *Indeed, we did this and the results are described in the paragraph "Depletion of ESCRT components in fzo-1(tm1133) animals with a block in autophagy results in embryonic lethality" in the Result section (see* ***pages 16/17****).*

Line 390. It is not clear why the 17 genes (out of 33) that were negative upon retesting for an autophagy phenotype were included in the final tally, nor whether they were part of the original set of 17 genes that were previously identified in published screens, or whether they came from the genes that arose from in silico and database analysis. This section would benefit from increased clarity, which may in fact mean providing less detail. For example, I would suggest leading off with your screening results, then referencing the other genes that were added due to demonstrated or predicted roles in the literature (which may have been false negatives in your screen) for a total of 143 out of the 299. This would be more direct and less confusing, IMHO. Of course, this is simply a suggestion. The data itself is wonderful.

Haeussler et al: *We agree with the reviewer that this was not very clear. We re-wrote this entire paragraph and now indicate in revised* ***Figure 4A*** *the genes that were found in our autophagy screen. The 'false negatives' that we missed in our screen but that have previously been shown to negatively regulate autophagy are, in our opinion, worth mentioning in this context as well.*

10. Figure 5. Another good reason to compare cleaved GFP to LGG::GFP rather than tubulin is the hars-1 result. If hars-1(RNAi) results in a 25% loss of GFP signal (as stated in the previous section), why is that result not reflected here? It appears as if the abundance of GFP is increased relative to tubulin. Could tubulin expression be suppressed even more dramatically? To me, this argues even more strongly that the ratio should be internally controlled.

Haeussler et al: *Please see comment #9.*

11. Figure 6. It is not clear to me that this data adds much to the paper, and it seems to be a weak way to end an otherwise solid story. Spg-7 lf results in a slight increase in autophagic foci, but much less compared to the mutants in mitochondrial dynamics. Hence, the starting line is different, and it is not surprising to me that they would react differently to additional stress. Bringing in the vps RNAi also confuses what should be a simple message: let-363(RNAi) does not suppress spg-7 lf mitoUPR induction as it

does mutants in mitochondrial dynamics. With that in mind, however, I think that the conclusion (ie. The induction of autophagy is sufficient to suppress mitoUPR induced by a block in mitochondrial dynamics but not by the loss of spg-7) ignores the 63% of your targets that you have shown negatively suppress autophagy, but that also suppress mitoUPR induction in the spg-7 lf mutant (ie. Unlike let-363). Since you are not showing the actual data for the spg-7 screen, I advocate for putting the let-363 result in a supplemental figure and ending with Figure 5. This would make for a stronger finish. Again, just a suggestion.

Haeussler et al: *We appreciate the reviewers comment. The revised manuscript has significantly changed and we now present lipidomics data. For this data, our results on drp-1 and spg-7 are critical and for this reason, we decided to not remove it.*

12. The link between MMP and atfs-1 is not completely understood, but it has been suggested that reduced MMP is insufficient to induce the mitoUPR. You may want to factor this into your model. Frankly, I am not convinced that the MMP is the deciding factor, and I'm not sure that it needs to be in order for this work to have value.

Haeussler et al: *Earlier this year, Rolland and colleagues showed that the efficiency of the mitochondrial import of ATFS-1 directly determines whether UPR^{mt} is induced or not (Rolland et al., 2019). In line with this study, increases in TMRE signal intensity and, hence, mitochondrial membrane potential upon autophagy induction most likely suppress fzo-1(lf)-induced UPR^{mt} by enhancing mitochondrial import of ATFS-1.*

Line 485. Please tone down ruling out mitophagy, since as you later acknowledge, there are pdr-1 independent mechanisms.

Haeussler et al: *We thank the reviewer for this comment. As suggested by reviewer 1, we now also tested fndc-1-dependent mitophagy. We found that fndc-1 dependent mitophagy also does not affect suppression of UPR^{mt} in this context (see **Figures 5C&5D**). The data is included in the revised manuscript on **page 17** and discussed on **page 24**.*

13. Page 22-23. I love your willingness to speculate in the discussion, but many of the ideas lack sufficient support to distinguish them from alternative competing ideas. It may be worth backing off just a little bit on specific mechanisms unless you are willing to test them, or can provide more concrete validation. Alternatively, there are simple questions that could be explored in more depth: what distinguishes fzo-1 and drp-1, which have different functions but similar outputs as regards mitoUPR and autophagy, or are they identical in all respects? Why does vps-37 RNAi induce mitophagy but not suppress fzo-1 lf?

Haeussler et al: *We now have support for our model that a defect in mitochondrial dynamics leads to specific metabolic changes, which are compensated by an increase in autophagy. Specifically, on **pages 21-23** of the Result section and in **Figures 7, S8, S9**, we are now presenting lipidomics data.*

14. Using both fzo-1 and drp-1 to implicate dynamics is powerful; did you look at eat-3 by chance? Being an inner membrane protein, I understand that a negative result could be difficult to interpret, but I did wonder…

Haeussler et al: *We did also look at eat-3(ad426) mutants and re-screened all candidates also in this mutant background. We found that ~78 % of the candidates reproduced in eat-3(ad426). However, as mentioned by the reviewer, being an inner membrane protein with functions other than regulating mitochondrial dynamics, this result is difficult to interpret. For this reason, we decided not to present it and focus on fzo-1 and drp-1.*

15. Your model suggests that defects in structural dynamics induce autophagy and the mitoUPR. Signaling must occur through a unidirectional pathway, since blocking or inducing the mitoUPR does not affect baseline autophagy. But suppressing ESCRT through RNAi (or let-363 or other negative regulators of autophagy) induces more autophagy and blocks the mitoUPR. This is consistent with a feedback mechanism. I think that all of these aspects of the model are well supported by the data. However, it is less clear to me that MMP is the discerning factor. The evidence is correlative and not nearly as convincing. Unless you can provide stronger support for the role of the MMP (mild upcoupling via low-dose FCCP treatment to establish causality?), I would suggest downplaying it.

Haeussler et al: *See comment about mitochondrial membrane potential (comment #12).*

Reviewer #3: In the manuscript entitled " Autophagy compensates for defects in mitochondrial dynamics", Haeussler and colleagues report an analysis of the mitochondrial dynamics using the nematode C. elegans. They first confirm that the depletion of FZO-1 and DRP-1, the respective homologs of mammalian MFN and DRP1, affects the mitochondrial network and show that both mutant induce mitochondrial UPR. The authors performed a genome wide RNAi screen to identify genes which depletion suppresses the UPRmit phenotype of the fzo-1 mutant. Among the candidates, they analyzed further the components of the ESCRT machinery. They show that in fzo-1 mutant there is an induction of autophagy flux and propose that it suppresses the UPRmit. A blockage of autophagy also induces UPRmit. By an RNAi approach, the authors claim that 143 genes are negative regulators of autophagy.
The results presented in the manuscript represent a substantial amount of work, in particular the genome-wide RNAi screen, but several major problems are weakening these results. The main problems concern the absence of clear biological questions driving the experiments, the miss of several controls and the fact that some conclusions are based on single observations and should be confirmed by another independent approach.

1) The result section starts by the description than fzo-1 and drp-1 mutants induce UPRmit. The two first pages of the results are discussing which reporter of UPRmit is good between a multi copy zcIs13 versus single copy bcSi9, but finally the authors use both strains. This section is not very informative or useful for the rest of the manuscript.

Haeussler et al: *The C. elegans field mostly uses the multi-copy transgene zcIs13. For this reason, we believe it is important to present our data on its draw-backs such as inter-animal variability and to present a thorough validation of our new single-copy transgene bcSi9.*

One aspect that is not addressed in the paper is the possibility of tissue-specific effects. It is known that the mitochondrial network is not similar in all C. elegans tissues? Are all the fzo-1 mutant tissues behaving similarly? In figure 1, close up are necessary to see in what tissues hsp-6::GFP and hsp-60::GFP are increased. It is essential to describe what happens in muscle or in epidermis, because these tissues are used to test mitochondrial fragmentation in Fig 2C or TMRE in Fig 2D,E, respectively. Moreover, the quantification of autophagic puncta has been performed within a sub population of epidermal cells (the seam cells)

Haeussler et al: *The reviewer addresses an important point, which in the revised manuscript, we have addressed in a number of ways.*

As suggested, we checked mitochondrial morphology in intestinal cells where hsp-6 and hsp-60 reporters are mostly expressed. We found that mitochondria are also fragmented in fzo-1(tm1133) in this tissue, and confirm that mitochondrial morphology remains unchanged upon induction of autophagy (see ***pages 11&14*** *and* ***Figure S3B****). We did not include close-ups of the UPR^{mt} reporters in Figure 1 but are stating in the Results section that the reporters are expressed in intestine and hypodermis. (To show whole worm images instead of close-ups is common practice in the C. elegans UPR^{mt} field.)*

Furthermore, TMRE stainings show mitochondrial morphology in hypodermal cells. We additionally included close-ups in these images to show that mitochondrial morphology is also fragmented in this tissue in fzo-1(tm1133) and remains unchanged upon induction of autophagy (see ***pages 11&14*** *and* ***Figure 2E****). We are testing TMRE staining intensity in the hypodermis because this tissue is easily accessible for the dye. Penetration becomes an issue in the case of deeper tissues.*

Finally, in C. elegans, the quantification of autophagic puncta is commonly done in hypodermal seam cells (please see methods papers Klionsky et al., 2016; Zhang et al., 2015). Additionally, we analyze induction of autophagy in intestinal cells using GFP::LGG-1 and are quantifying whole worm extracts for cleaved GFP levels on western blots.

2) Using an RNAi approach the authors show that the depletion of several ESCRT components suppresses UPRmit in fzo-1 mutant.
In figure 1, some controls are missing. All RNAi should be also shown in the wild-type background and not only in fzo-1(tm1133).

Haeussler et al: *We thank the reviewer for this comment, which we have addressed. We now also tested RNAi against ESCRT components in wild-type animals and found that atfs-1(RNAi), vps-4(RNAi) and vps-20(RNAi) suppress the reporter expression to a certain degree (see revised* ***Figure S2A****), however the suppression in these cases is rather mild. bcSi9 expression in the wild-type background reflects baseline hsp-6 expression. (We would like to point out that non-stress related, baseline expression of hsp-6 has important functions in mitochondrial*

import and therefore differs in function from the role of hsp-6 expression in the mitochondrial stress response examined in fzo-1(tm1133).) The data is included in the Result section on ***page 9****.*

What is the efficiency of RNAi ? It has been shown that RNAi against ESCRT are not always recapitulating mutant phenotypes (ref. 36), so mutants could be used to confirm the RNAi phenotype. This is important for ESCRT components which depletion does not present any effect on fzo-1 phenotypes (vps-20 and vps-36).

Haeussler et al: *We agree with the reviewer in principal, but we do not think that analyzing ESCRT mutants would add additional information to the manuscript. Importantly, analyzing ESCRT mutants is challenging for the following reasons. First, most of the ESCRT mutants are lethal and arrest before the L4 larval stage, the stage at which we perform all of our assays. Second, the few ESCRT mutants that are viable post L4 stage have to be balanced in order to be maintained. Therefore, the homozygous mutants may be maternally rescued, which would make any negative results very difficult to interpret. Finally, the connection between ESCRT depletion and autophagy induction was subject to a previous report (Djeddi et al., 2012). We now describe a functional connection between the induction of autophagy and suppression of fzo-1(lf)-induced* UPR^{mt} *via the ESCRTs.*

3) From figure 2, the authors conclude that ESCRT depletion does not improve mitochondrial fragmentation but increases membrane potential. The mitochondrial fragmentation is shown in muscle and the mitochondrial dye TMRE is used in epidermis to analyze the mitochondrial potential membrane For Fig 2C, pictures of mitoGFP in ESCRT(RNAi) alone should be shown.

Haeussler et al: *We thank the reviewer for this suggestion. We now also knocked-down vps-4, vps-20, let-363 and atfs-1 in wild-type animals expressing mitoGFP in body wall muscle cells. We did not see a difference in mitochondrial morphology in these experiments (see* ***Figure S3A****). The data is included on* ***pages 11 and 14****.*

TMRE data are not really convincing because alone it does not allow deciphering between a change in the membrane potential and a change in the mitochondrial mass. One possibility is to combine TMRE with another mitochondrial dye independent of MMP, or a GFP marker of the mitochondria.

Haeussler et al: *We thank the reviewer for this comment, because it made us realize that we had not explained well enough how we quantify the TMRE signal. The 'TMRE fluorescence intensity per mitochondrial area' was measured by segmentation of mitochondria in the recorded images, followed by quantification of fluorescence intensity only in the segmented areas i.e. the mitochondria. A schematic describing the workflow is now presented in* ***Figure S4*** *and the Result section was modified accordingly on* ***page 11****. Additionally, we included close-ups of TMRE stainings in the hypodermis (****Figure 2E****), which show that mitochondrial morphology is also unchanged upon autophagy induction in the hypodermis.*

It is surprising that fzo-1 mutant have almost no staining with TMRE in Fi g2D. The measure of the oxygen consumption rate should be also perform to confirm these data.

Haeussler et al: *We thank the reviewer for this suggestion. Oxygen consumption rate was published earlier for fzo-1(tm1133) where basal OCR was unchanged and maximal OCR was found to be decreased, compared to wild type (Luz et al., 2015). The fact that basal OCR was unchanged in fzo-1(tm1133) may be explained by compensatory mechanisms (e.g. metabolic shift towards glycolysis) that produce ATP independent of the electron transport chain and outside of mitochondria.*

*We tried to address this earlier in the project and planned to measure mitochondrial function using a Clark oxygen electrode in collaboration with Andrew Wojtovich (University of Rochester Medical Center). These experiments require high amounts of staged worms (~ 0.5 million worms) for isolation of mitochondria. Unfortunately, it is not possible to obtain those high number of staged fzo-1(tm1133) animals due to their variable developmental delays. We additionally tried to measure OCR using the Seahorse Analyzer XF in collaboration with Fabiana Perocchi (Helmholtz Centre, Neuherberg Germany) but could not establish a reliable protocol and got inconsistent results in this assay (see **reviewer#1, comment #1** and **Figures R1-R5**).*

4) The authors convincingly show that there is an increase of autophagic flux in fzo-1 animals. Fig 3C is too small and data are not quantified.

Haeussler et al: *Images of embryos expressing sqst-1::gfp are commonly not quantified in the autophagy field in C. elegans since SQST-1::GFP dots only appear upon a block in autophagy. Moreover, the data in Figure 3C provides additional information to exclude a block in autophagy in fzo-1(tm1133) animals, which is consistent with the data in Figure 3D where we show that cleaved GFP levels in fzo-1(tm1133) are increased compared to wild type.*

ESCRT(RNAi); fzo-1 animals present a further increase in autophagy. It supports previously published data showing that ESCRT depletion induces autophagic flux but does not prove that there is a causal link between FZO-1 and ESCRT. In Fig 3E, controls for ESCRT(RNAi) alone are not shown

Haeussler et al: *We do not want to establish any causality between ESCRTs and fzo-1 but are focusing on the link between induction of autophagy and suppression of UPR^{mt}. As suggested by the reviewer, we included wild-type controls for the P_{lgg-1}gfp::lgg-1 reporter upon ESCRT(RNAi) (see **Figure S2B**) confirming previously published results (Djeddi et al., 2012). The data is now included on **page 12** in the Result section of the revised manuscript.*

5) Using let-363(RNAi) (TOR homolog), the authors conclude that an autophagy induction suppresses the mitochondrial UPR in fzo-1 mutant. mTOR is involved in regulating autophagy but also in many others processes that can affect homeostasis and let-363 animals have many phenotypes. It could be useful to show that a blockage of autophagy flux (atg-5, atg-7 ...) in let-363 mutant blocks the effect on UPRmit.

Haeussler et al: *We thank the reviewer for this suggestion, which we tried to address. Unfortunately, we were unable to perform this experiment because let-363(h111) mutants alone arrest during the L3 stage. Furthermore, we suspect that the let-363(h111); fzo-1(tm1133)*

double mutant will arrest at an even earlier stage of development and that additional RNAi treatment in order to block autophagy will worsen the phenotype of these animals. Furthermore, we also show that let-363(RNAi) does suppress the $P_{ges\text{-}1}$::gfp reporter to some degree, which shows that the suppression seen for the bcSi9 reporter most likely is the result of a combination of the induction of autophagy and the attenuation of translation by let-363(RNAi) (as also shown for hars-1(RNAi)).

6) The paragraph "Depletion of ESCRT components in fzo-1(tm1133) animals with a block in autophagy results in embryonic lethality" is not informative because the authors could not test whether the increase of autophagic flux in ESCRT depleted fzo-1 animals is responsible of the improvement of mitochondrial UPR. Then, they tested RNAi against one pathway involved in mitophagy. However, the authors have not demonstrated that there is mitophagy occurring in fzo-1 mutants or ESCRT(RNAi); fzo-1. Moreover, there are others mitophagic pathways parallel to PDR-1 which have not been tested (Fundc1). The conclusion of this section is rather weak.

Haeussler et al: *The reviewer addresses an important point. Unfortunately, in C. elegans, the discrimination between general autophagy and selective autophagy /mitophagy is not possible due to the lack of reliable mitophagy specific assays. For this reason, we cannot distinguish between general autophagy and mitophagy in fzo-1(tm1133). However, we wanted to test if mitophagy rather than general autophagy is responsible for the suppression of fzo-1(lf)-induced UPR^{mt} upon ESCRT(RNAi) and therefore tested one of the few known mitophagy genes in C. elegans, pdr-1. As suggested by the reviewers, we include data showing that the block of fndc-1-dependent mitophagy also does not prevent the suppression of fzo-1(lf)-induced UPR^{mt} upon ESCRT(RNAi) (see revised* ***Figures 5C and 5D****). In summary, we think that this paragraph is still of interest since neither pdr-1- nor fndc-1-dependent mitophagy has a role in the suppression of fzo-1(lf)-induced UPR^{mt} upon ESCRT(RNAi) and therefore we speculate that general autophagy rather than mitophagy is responsible for the suppression of fzo-1(lf)-induced UPR^{mt} upon ESCRT(RNAi). The new data is included in the Result section on* ***page 17*** *and in the Discussion section on* ***page 24*** *of the revised manuscript.*

7) The authors show data indicating that blocking autophagy induces UPRmit but does not further increase UPRmit in fzo-1. It would be interesting to test whether it is due to an accumulation of damages that are not removed or an active induction. Conversely depleting ATFS-1 does not modify autophagic response of fzo-1 animals. Unfortunately, the effect of overexpression could not been studied.

Haeussler et al: *The reviewer raises a number of very interesting points. Indeed, we added new data to the manuscript showing that a block in mitochondrial dynamics leads to changes in lipid metabolism which are partially compensated by the induction of autophagy (see below; comment #9). However, to determine how a block in autophagy leads to UPR^{mt} induction is beyond the scope of our current manuscript.*

8) The last part of the manuscript presents a combination of data mining and RNAi to identify

143 candidates that the authors qualify of "negative regulators of autophagy". This is affirmed on the observation that RNAi of these genes results in increase of GFP::LGG-1 dots in the seam cells and no accumulation of SQST-1. Six genes among the 143 candidates have been further tested for autophagy induction after RNAi depletion. This approach shows that autophagy is potentially increased upon RNAi treatments, but it could be a very indirect consequence. Demonstrating that the overexpression of these proteins decrease the autophagic flux is necessary to conclude that these candidates are bona fide negative regulators.

Haeussler et al: *We thank the reviewer for this comment. We are defining these genes as "negative regulators of autophagy" from a geneticist's point of view because autophagy is induced when these genes are knocked-down. We completely agree with the reviewer in that knock-down of these genes most likely indirectly induces autophagy.*

9) In the discussion, an interesting hypothesis proposes that the modifications of mitochondrial networks (fzo-1 and drp-1) induce the UPRmit and then a metabolic change. Do the authors have any data about a potential metabolic shift?

Haeussler et al: *We indeed addressed this point and found different lipid compositions in fzo-1(tm1133), drp-1(tm1108) and spg-7(ad2249) mutants (see **Figure S8**). We then induced autophagy in fzo-1(tm1133) mutants in order to see whether the observed changes in lipid composition can be reverted. We now included this data and show that especially the levels of triacylglycerols are changed in fzo-1, as compared to wild type, and that this is at least partially reverted upon induction of autophagy (see **Figures 7 and S9**). The data is included on **pages 21-23** of the revised manuscript.*

Authors should discuss the potential mechanism(s) of action of ESCRTs on autophagy and mitochondrial dynamics.

Haeussler et al: *We thank the reviewer for this comment. Depletion of ESCRT components could generally compromise the efficiency of the endosomal system and may eventually result in amino acid deprivation and therefore induce autophagy. Without elevated autophagy, amino acid starvation may even be more drastic, making the animals unable to produce sufficient amount of proteins, which could ultimately lead to lethality. However, since the action of ESCRT on autophagy is not the main focus of our manuscript and the discussion is rather lengthy, we did not discuss this topic in any detail. The effects of induction of autophagy on lipid metabolism and how we think that this might work mechanistically, is now discussed in more detail.*

ESCRT defective mutant triggers an adaptative autophagic flux, but recent data have shown that ESCRT are involved in autophagosome closure (Takahashi Nature com 2018; Zhou JCB 2019). These references should be added and discussed.

Haeussler et al: *We are now including the above-mentioned papers (see **page 5**).*

Minor points

For laudable pedagogic reason the authors propose a combination of C. elegans and human names for genes and proteins, but it does not respect the C. elegans nomenclature and in particular cases complicate the comprehension (Phsp-6 mtHSP70gfp). C. elegans Vps-27 official name is Hgrs. LGG-1 is the homolog of the GABARAP family rather than the LC3

Haeussler et al: *We thank the reviewer for pointing this out. The gene name of vps-27 was changed to hgrs-1 throughout the manuscript and figures and the LGG-1 homolog was also changed to GABARAP. C. elegans nomenclature is, in our opinion, not disrespected by adding the homologs directly in the text.*

Appendix

We included all figures that contain experimental data of the revision process which is not included in the manuscript.

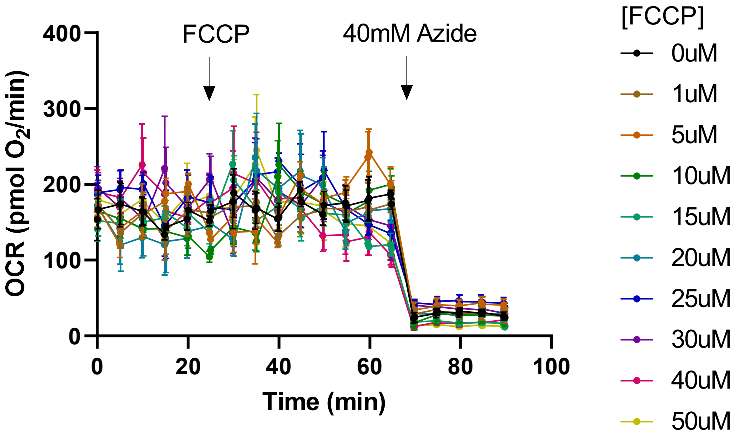

Figure R1: FCCP titration in Seahorse Analyzer XF96 from 0 µM to 50 µM. Each FCCP concentration was tested in three wells, each containing 25 L4 larvae of the *fzo-1(tm1133)* genotype. OCR was measured in 5 cycles before and 8 cycles after FCCP injection. Finally, 40 mM Sodium Azide were injected and OCR was measured for 5 cycles.

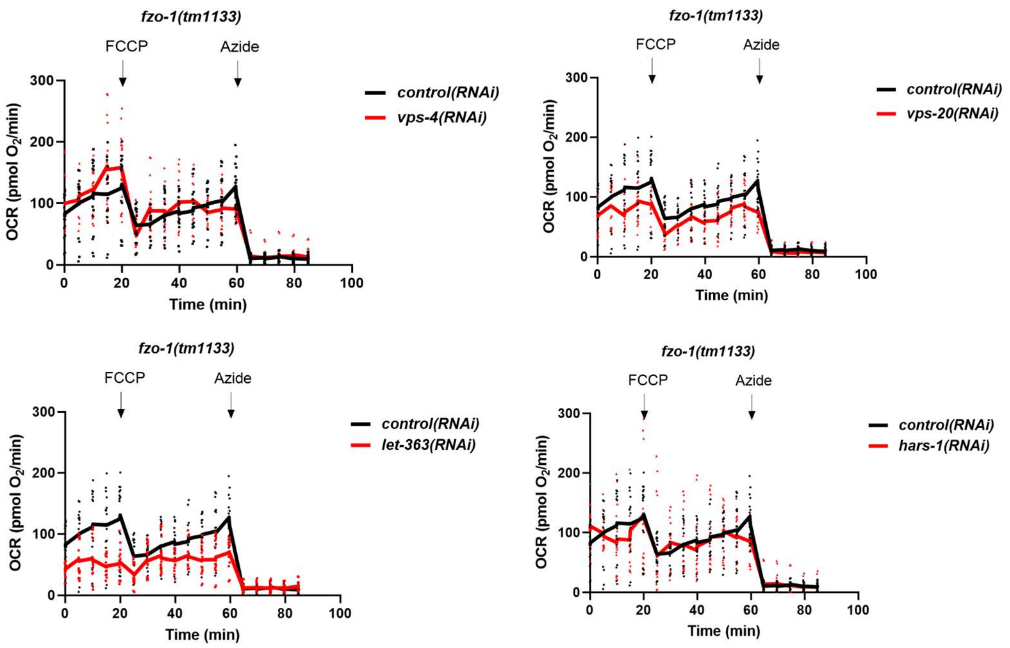

Figure R2: OCR measurements in *fzo-1(tm1133)* upon induction of autophagy. L4 larvae were subjected to respective RNAi and 25 L4 larvae of the following F1 generation were used to measure OCR in octuplicates in the Seahorse Analyzer XF96.

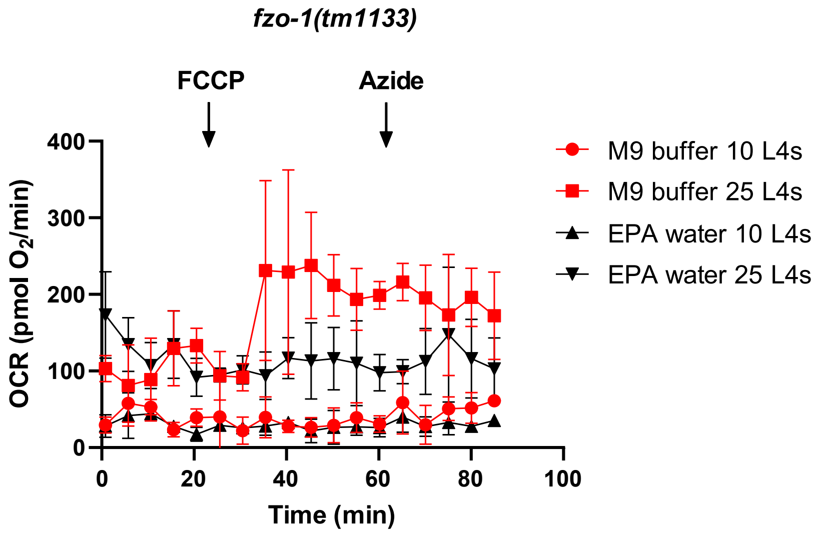

Figure R3: OCR measurements in *fzo-1(tm1133)* using different buffers. Measurement of OCR of L4 larvae in M9 and "EPA water" in the Seahorse Analyzer XF96.

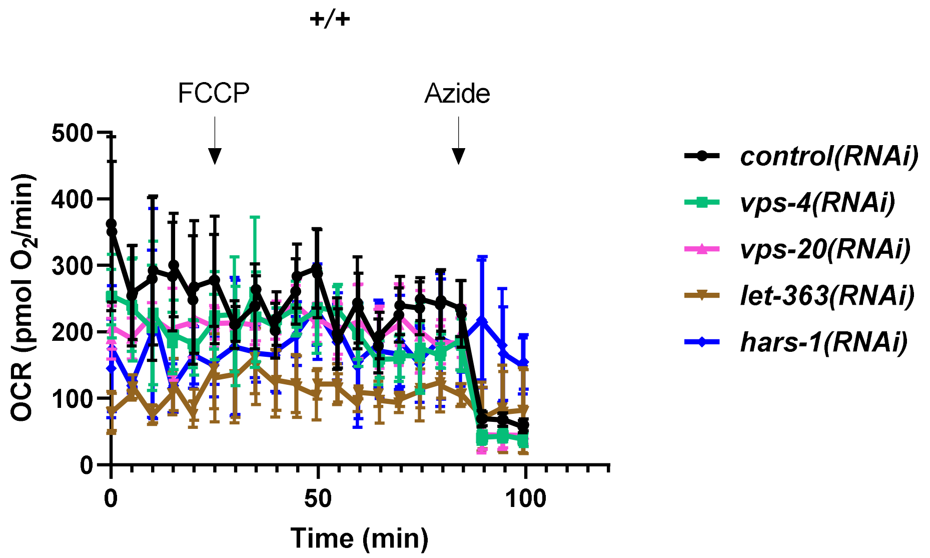

Figure R4: OCR measurements in +/+ after induction of autophagy. Measurement of OCR of L4 larvae in M9 after knock-down of respective genes in the Seahorse Analyzer XF96.

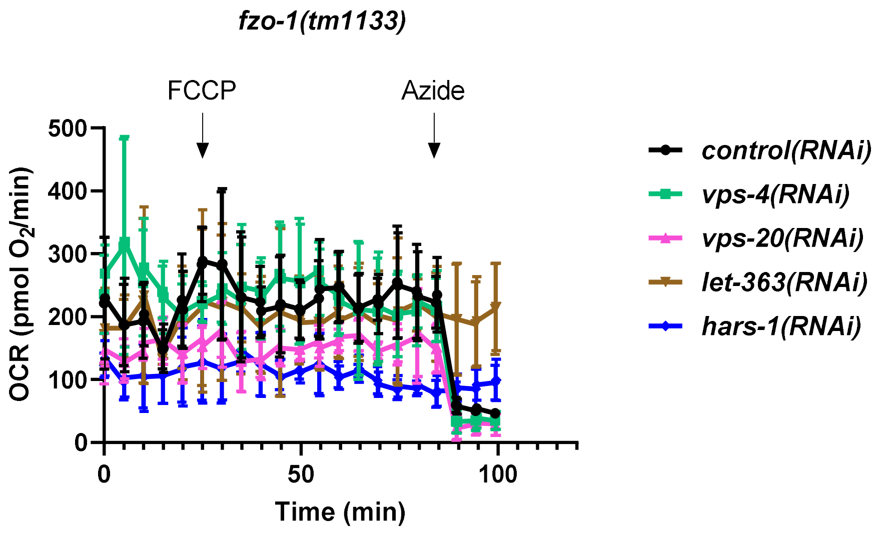

Figure R5: OCR measurements in *fzo-1(tm1133)* after induction of autophagy. Measurement of OCR of L4 larvae in M9 after knock-down of respective genes in the Seahorse Analyzer XF96.

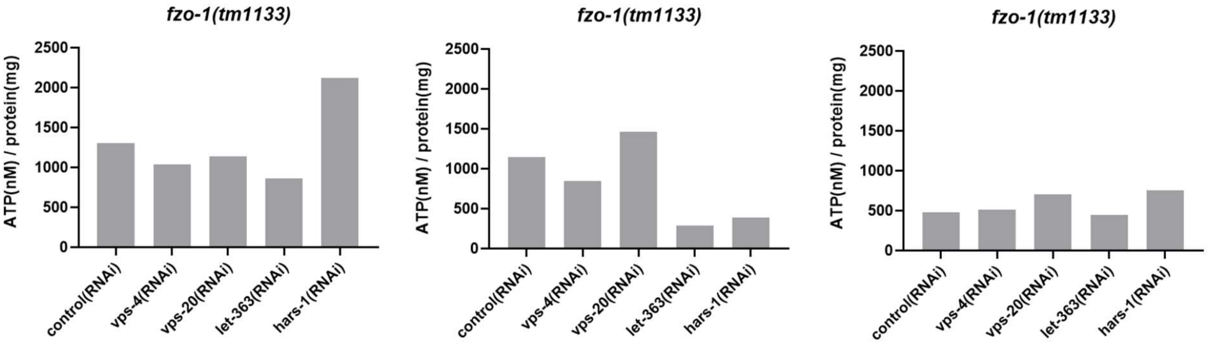

Figure R6: Total ATP measurements in *fzo-1(tm1133)* upon autophagy induction using a luciferase assay. L4 larvae were subjected to respective RNAi and 100 L4 larvae of the following F1 generation were used to determine total ATP levels as described (Palikaras and Tavernarakis, 2016). Data of three independent experiments are shown.

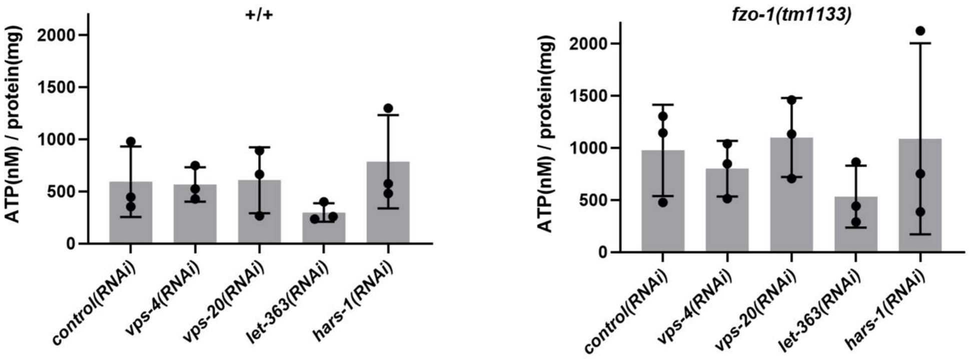

Figure R7: Total ATP measurements in wild type and *fzo-1(tm1133)* upon autophagy induction using a luciferase assay. L4 larvae were subjected to respective RNAi and 100 L4 larvae of the following F1 generation were used to determine total ATP levels as described (Palikaras and Tavernarakis, 2016). n = 3.

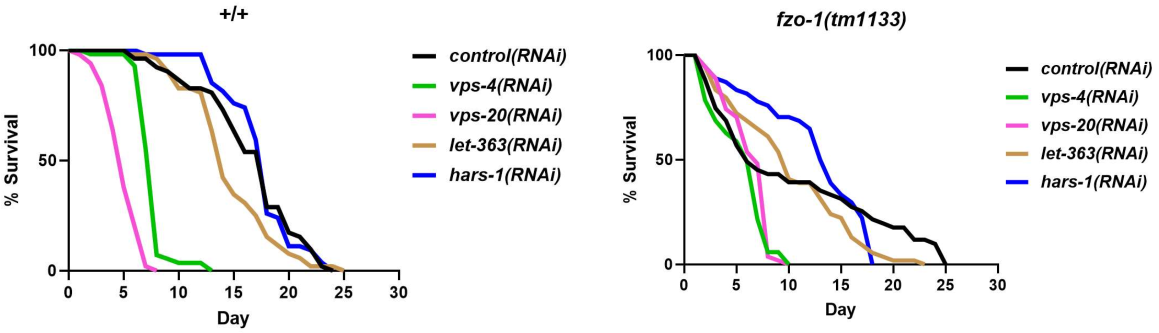

Figure R8: Life span analysis in wild-type and *fzo-1(tm1133)* animals upon induction of autophagy. For each condition, ≥ 50 animals of the F1 generation after knock-down of respective genes were used for life span analysis. L4 stage was set as d0 of the life span experiment. n = 1

References

Djeddi, A., Michelet, X., Culetto, E., Alberti, A., Barois, N., and Legouis, R. (2012). Induction of autophagy in ESCRT mutants is an adaptive response for cell survival in *C. elegans*. Journal of cell science *125*, 685-694.

Hansen, M., Taubert, S., Crawford, D., Libina, N., Lee, S.-J., and Kenyon, C. (2007). Lifespan extension by conditions that inhibit translation in Caenorhabditis elegans. Aging cell *6*, 95-110.

Johnson, D., and Nehrke, K. (2010). Mitochondrial Fragmentation Leads to Intracellular Acidification in Caenorhabditis elegans and Mammalian Cells. Molecular Biology of the Cell *21*, 2191-2201.

Kennedy, B.K., and Kaeberlein, M. (2009). Hot topics in aging research: protein translation, 2009. Aging cell *8*, 617-623.

Klionsky, D.J., Abdelmohsen, K., Abe, A., Abedin, M.J., Abeliovich, H., Acevedo Arozena, A., Adachi, H., Adams, C.M., Adams, P.D., Adeli, K.*, et al.* (2016). Guidelines for the use and interpretation of assays for monitoring autophagy (3rd edition). Autophagy *12*, 1-222.

Koopman, M., Michels, H., Dancy, B.M., Kamble, R., Mouchiroud, L., Auwerx, J., Nollen, E.A.A., and Houtkooper, R.H. (2016). A screening-based platform for the assessment of cellular respiration in Caenorhabditis elegans. Nature Protocols *11*, 1798-1816.

Luz, A.L., Rooney, J.P., Kubik, L.L., Gonzalez, C.P., Song, D.H., and Meyer, J.N. (2015). Mitochondrial Morphology and Fundamental Parameters of the Mitochondrial Respiratory Chain Are Altered in Caenorhabditis elegans Strains Deficient in Mitochondrial Dynamics and Homeostasis Processes. PloS one *10*, e0130940.

Palikaras, K., and Tavernarakis, N. (2016). Intracellular Assessment of ATP Levels in Caenorhabditis elegans. Bio Protoc *6*, e22048.

Rolland, S.G., Schneid, S., Schwarz, M., Rackles, E., Fischer, C., Haeussler, S., Regmi, S.G., Yeroslaviz, A., Habermann, B., Mokranjac, D.*, et al.* (2019). Compromised Mitochondrial Protein Import Acts as a Signal for UPR^{mt}. Cell reports *28*, 1659-1669.e1655.

Vellai, T., Takacs-Vellai, K., Zhang, Y., Kovacs, A.L., Orosz, L., and Müller, F. (2003). Influence of TOR kinase on lifespan in C. elegans. Nature *426*, 620-620.

Zhang, H., Chang, J.T., Guo, B., Hansen, M., Jia, K., Kovacs, A.L., Kumsta, C., Lapierre, L.R., Legouis, R., Lin, L.*, et al.* (2015). Guidelines for monitoring autophagy in *Caenorhabditis elegans*. Autophagy *11*, 9-27.

Zhou, B., Kreuzer, J., Kumsta, C., Wu, L., Kamer, K.J., Cedillo, L., Zhang, Y., Li, S., Kacergis, M.C., Webster, C.M.*, et al.* (2019). Mitochondrial Permeability Uncouples Elevated Autophagy and Lifespan Extension. Cell *177*, 299-314.e216.
