# Supplementary material for: Autophagy compensates for defects in mitochondrial dynamics
Source: PLoS Genet. 2020 Mar 19;16(3):e1008638. doi: 10.1371/journal.pgen.1008638 (PMC7135339; doi:10.1371/journal.pgen.1008638)
Supplement: Attachment — Submitted filename: PLoS Genetic July 2019 review.docx [file pgen.1008638.s013.docx]

This is a solid piece of work describing an unexpected signaling axis between regulators of mitochondrial dynamics, the mitochondrial unfolded protein response, and autophagy. The approach is generally sophisticated, rigorous, well-executed, and appropriate for PLoS Genetics, and the results will contribute significantly to the field. I want to be clear that I am very enthusiastic regarding this story. I used this manuscript to train one of my postdoctoral fellows in the review process, which contributed to its length. Within that context, I hope that the criticisms detailed below will help to make the manuscript even stronger. I have listed a few major points, the first of which should be addressed experimentally, followed by minor points and suggestions to improve clarity and flow, which may be addressed at the author’s discretion but should not require additional experiments.

Major points:

1. Line 240. The TMRE result needs to be interpreted with caution. Obviously, there is much less staining in *fzo-1(tm1133)* mutants, consistent with fragmentation having been associated previously with loss of MMP. However, while ESCRT(RNAi) suppresses this loss, the fact that suppression is modest at best and is extremely variable warrants careful consideration of TMRE’s dye loading, distribution, and equilibrium, which may not be identical in worms of differing genotypes. Moreover, mitochondrial content may differ, as well – which may be quite relevant, as *atfs-1* dependent mitoUPR induction may drive a mitogenesis program upon fragmentation. In addition, TMRE can be used in both quenching and nonquenching modes, which depends upon its concentration and causes changes in mito membrane potential to present oppositely. To specifically address these issues, I would suggest querying mito::nuclear DNA ratios using qPCR, applying MitoTracker Green as a non-MMP dependent assessment of mito content, ascertaining that you are working in non-quench mode, and employing a separate measure of membrane potential to confirm the TMRE result for several of the most relevant target genes. These techniques in parallel will enhance scientific rigor and greatly strengthen the conclusion.
2. Line 307. The claim that *let-363*(RNAi) increased mito membrane potential (MMP) in the *fzo-1(tm1133)* mutant is not supported by the data. Even though there’s a 19% increase in signal, it is not statistically significant. This may be concerning, since *let-363* is marketed as being a “control” regulator that ties together autophagy and MMP.
3. Line 413. The logic of saying that the *hars-1*(RNAi) phenotype supports a role for genes in the GO-terms “Translation” and “Ribosomal Biogenesis” having a beneficial effect on MMP is faulty. Not only in the *hars-1*(RNAi) effect on MMP not statistically significant, there is no evidence to support this conclusion except that *hars-1* also falls under these GO-terms. This conclusion should be removed or undergo a severe reduction in strength to reflect the very weak support.

Minor points:

4. Line 83. Strictly speaking, ATFS-1 does not translocate to the nucleus (from the mito), even though often referred to in the context of retrograde mito-nuclear signal transduction. For clarity, I would encourage “targeting of ATFS-1 is redirected from the mito to the nucleus” or some variant thereof. This comment applies as well to similar statements in the discussion.
5. Lines 155-186. This entire section is somewhat distracting. It is not intuitive as to why the multicopy transgene would be better for screening, particularly since the MosSCI transgene is used for screening later in the paper, as well as all of the validation approaches. Regardless, the authors may want to consider holding off on introducing the MosSCI allele until they actually use it. Truth-be-told, the Results section could begin with line 188 without losing any impact, and I think this would make for a tighter story.
6. General comment. Please double-check that you are consistent in the use of past versus present tense.
7. Line 220. It is likely worth reiterating here for readers outside the field that *hsp-60* is an alternative *atfs-1* dependent mitoUPR target gene. It is also worth considering that *hsp-6* and *hsp-60*, while canonical readouts for the mitoUPR, may not accurately reflect all aspects of its induction (take for example the difference between the *atfs-1* gf mutant and *spg-7* RNAi, even though they both induce the hsps).
8. Figure 2F. The data shows that *vps* RNAI partly suppresses the *fzo-1* lf mutant effect on MMP. Do any of these RNAi treatments have an effect on MMP in a wildtype background?
9. Figure 3D, E. I think that it would be more appropriate to assess the ratio of cleaved GFP to GFP::LC3 rather than to tubulin. Is there a good argument for using tubulin?
10. Why is there no quantitation of the LGG foci in Figure 3E?
11. Did you test whether *unc-51* suppresses the effect of *vps* RNAi in the *fzo-1* lf mutant? If so, it is worth mentioning.
12. Line 390. It is not clear why the 17 genes (out of 33) that were negative upon retesting for an autophagy phenotype were included in the final tally, nor whether they were part of the original set of 17 genes that were previously identified in published screens, or whether they came from the genes that arose from in silico and database analysis. This section would benefit from increased clarity, which may in fact mean providing less detail. For example, I would suggest leading off with your screening results, then referencing the other genes that were added due to demonstrated or predicted roles in the literature (which may have been false negatives in your screen) for a total of 143 out of the 299. This would be more direct and less confusing, IMHO. Of course, this is simply a suggestion. The data itself is wonderful.
13. Figure 5. Another good reason to compare cleaved GFP to LGG::GFP rather than tubulin is the *hars-1* result. If *hars-1*(RNAi) results in a 25% loss of GFP signal (as stated in the previous section), why is that result not reflected here? It appears as if the abundance of GFP is increased relative to tubulin. Could tubulin expression be suppressed even more dramatically? To me, this argues even more strongly that the ratio should be internally controlled.
14. Figure 6. It is not clear to me that this data adds much to the paper, and it seems to be a weak way to end an otherwise solid story. *Spg-7* lf results in a slight increase in autophagic foci, but much less compared to the mutants in mitochondrial dynamics. Hence, the starting line is different, and it is not surprising to me that they would react differently to additional stress. Bringing in the *vps* RNAi also confuses what should be a simple message: *let-363*(RNAi) does not suppress *spg-7* lf mitoUPR induction as it does mutants in mitochondrial dynamics. With that in mind, however, I think that the conclusion (ie. the induction of autophagy is sufficient to suppress mitoUPR induced by a block in mitochondrial dynamics but not by the loss of *spg-7*) ignores the 63% of your targets that you have shown negatively suppress autophagy, but that also suppress mitoUPR induction in the *spg-7* lf mutant (ie. unlike *let-363*). Since you are not showing the actual data for the *spg-7* screen, I advocate for putting the *let-363* result in a supplemental figure and ending with Figure 5. This would make for a stronger finish. Again, just a suggestion.
15. The link between MMP and *atfs-1* is not completely understood, but it has been suggested that reduced MMP is insufficient to induce the mitoUPR. You may want to factor this into your model. Frankly, I am not convinced that the MMP is the deciding factor, and I’m not sure that it needs to be in order for this work to have value.
16. Line 485. Please tone down ruling out mitophagy, since as you later acknowledge, there are *pdr-1* independent mechanisms.
17. Page 22-23. I love your willingness to speculate in the discussion, but many of the ideas lack sufficient support to distinguish them from alternative competing ideas. It may be worth backing off just a little bit on specific mechanisms unless you are willing to test them, or can provide more concrete validation. Alternatively, there are simple questions that could be explored in more depth: what distinguishes *fzo-1* and *drp-1*, which have different functions but similar outputs as regards mitoUPR and autophagy, or are they identical in all respects? Why does *vps-37* RNAi induce mitophagy but not suppress *fzo-1* lf?
18. Using both *fzo-1* and *drp-1* to implicate dynamics is powerful; did you look at *eat-3* by chance? Being an inner membrane protein, I understand that a negative result could be difficult to interpret, but I did wonder…
19. Your model suggests that defects in structural dynamics induce autophagy and the mitoUPR. Signaling must occur through a unidirectional pathway, since blocking or inducing the mitoUPR does not affect baseline autophagy. But suppressing ESCRT through RNAi (or *let-363* or other negative regulators of autophagy) induces more autophagy and blocks the mitoUPR. This is consistent with a feedback mechanism. I think that all of these aspects of the model are well supported by the data. However, it is less clear to me that MMP is the discerning factor. The evidence is correlative and not nearly as convincing. Unless you can provide stronger support for the role of the MMP (mild upcoupling via low-dose FCCP treatment to establish causality?), I would suggest downplaying it.
